# Host-Associated Genomic Features of the Novel Uncultured Intracellular Pathogen *Ca.* Ichthyocystis Revealed by Direct Sequencing of Epitheliocysts

**DOI:** 10.1093/gbe/evw111

**Published:** 2016-05-10

**Authors:** Weihong Qi, Lloyd Vaughan, Pantelis Katharios, Ralph Schlapbach, Helena M.B. Seth-Smith

**Affiliations:** ^1^Functional Genomics Center Zurich, University of Zurich, Switzerland; ^2^Vetsuisse Faculty, Institute for Veterinary Pathology, University of Zurich, Switzerland; ^3^Hellenic Center for Marine Research, Institute of Marine Biology, Biotechnology and Aquaculture, Heraklion, Greece

**Keywords:** mini-metagenome, genome reduction, metabolic pathways, gene duplication-diversification, virulence, epitheliocystis

## Abstract

Advances in single-cell and mini-metagenome sequencing have enabled important investigations into uncultured bacteria. In this study, we applied the mini-metagenome sequencing method to assemble genome drafts of the uncultured causative agents of epitheliocystis, an emerging infectious disease in the Mediterranean aquaculture species gilthead seabream. We sequenced multiple cyst samples and constructed 11 genome drafts from a novel beta-proteobacterial lineage, *Candidatus* Ichthyocystis. The draft genomes demonstrate features typical of pathogenic bacteria with an obligate intracellular lifestyle: a reduced genome of up to 2.6 Mb, reduced G + C content, and reduced metabolic capacity. Reconstruction of metabolic pathways reveals that *Ca*. Ichthyocystis genomes lack all amino acid synthesis pathways, compelling them to scavenge from the fish host. All genomes encode type II, III, and IV secretion systems, a large repertoire of predicted effectors, and a type IV pilus. These are all considered to be virulence factors, required for adherence, invasion, and host manipulation. However, no evidence of lipopolysaccharide synthesis could be found. Beyond the core functions shared within the genus, alignments showed distinction into different species, characterized by alternative large gene families. These comprise up to a third of each genome, appear to have arisen through duplication and diversification, encode many effector proteins, and are seemingly critical for virulence. Thus, *Ca*. Ichthyocystis represents a novel obligatory intracellular pathogenic beta-proteobacterial lineage. The methods used: mini-metagenome analysis and manual annotation, have generated important insights into the lifestyle and evolution of the novel, uncultured pathogens, elucidating many putative virulence factors including an unprecedented array of novel gene families.

## Introduction

The study of obligate intracellular bacteria is often complicated by the difficulties of bringing them into culture. In these cases, where genetic and phenotypic studies are impossible, genome sequencing offers an opportunity to gain insights into the bacterial lifestyle and evolution. This technique is, however, often hindered by the availability of suitable quantities of sufficiently pure DNA to be able to sequence and assemble a full genome. Several techniques developed in recent years have enabled DNA sequencing from challenging samples (Raghunathan et al. 2005; Bos et al. 2011; Seth-Smith et al. 2013; Christiansen et al. 2014; NEBNext Microbiome DNA Enrichment Kit, NEB, Ipswich, MA and the QIAamp DNA Microbiome Kit, Qiagen, Hilden, Germany). In the pursuit of genomic information on novel pathogens, methods for the sequencing of single bacterial cells, and computational reconstruction of single genomes from mini-metagenomes have revolutionized microbial genomics (Lasken 2012; Lasken and McLean 2014; McLean and Lasken 2014). Such direct sequencing methods have been used to investigate marine symbionts (Hallam et al. 2006; Siegl et al. 2011; Schofield et al. 2015).

Intracellular bacteria have been described in many bacterial clades, as mutualists and pathogens. Many genomes of obligate intracellular bacteria have been sequenced, most commonly belonging to the alpha- and gamma-proteobacteria and chlamydial phylogenetic clades (Toft and Andersson 2010). Beta-proteobacteria with similar lifestyles have been reported in various host systems (Heinz et al. 2007; Hahn et al. 2009; Matsuo et al. 2010; Nakabachi et al. 2013), but only a few genomes are available (Lackner et al. 2011; Fujimura et al. 2014). The genomes of host-associated bacteria tend to show a reduction in metabolic capacity (Moran 2003), as they scavenge many resources from the host cell. In addition, they require the ability to interact with, adhere to, and influence to host cell, usually through the use of secretion systems (Toft and Andersson 2010).

We recently described a novel genus of the uncultured intracellular pathogen, *Candidatus* Ichthyocystis, using microscopic and molecular techniques (Seth-Smith et al. 2016). The two beta-proteobacterial species identified, named *Ca*. Ichthyocystis hellenicum and *Ca*. I. sparus, are causative agents of the disease epitheliocystis in gilthead seabream (*Sparus aurata*) in the Mediterranean (Seth-Smith et al. 2016). Gilthead seabream is the dominant species in Mediterranean aquaculture (FAO 2012) and epitheliocystis is an emerging disease, posing an economic risk to this industry. Through their function filtering water, gill epithelia are constantly exposed to environmental microbes and are a target organ for pathogens. In this disease, cysts form on the gill epithelia, causing inflammation, epithelial hyperplasia, a fusion of lamellae, and reduced ability to uptake oxygen (Seth-Smith et al. 2016). While epitheliocystis is commonly associated with chlamydial bacteria (Draghi et al. 2004, 2007; Karlsen et al. 2008; Schmidt-Posthaus et al. 2012; Fehr et al. 2013; Steigen et al. 2013; Stride, Polkinghorne, Miller, Groff, et al. 2013; Stride, Polkinghorne A, Miller TL and Nowak 2013; Guevara Soto et al. 2016), there is increasing evidence that beta- and gamma-proteobacteria can produce similar pathologies (Toenshoff et al. 2012; Mendoza et al. 2013; Contador et al. 2015; Katharios et al. 2015; Seth-Smith et al. 2016).

In this study, we applied the mini-metagenome sequencing method to study samples of the new genus *Ca*. Ichthyocystis, improving and extending the previous preliminary genomic analysis (Seth-Smith et al. 2016). Infected gill material preserved in RNALater or ethanol was used as the source of microdissected epitheliocysts for sequencing. Resulting sequence assemblies containing data from bacteria and host were computationally sorted, and we present a thorough analysis of 11 nearly complete draft genome sequences, two of which have been manually annotated as reference genomes for each species. This genus displays a compact core genome with reduced metabolic functionality, along with massive gene family duplication and diversification providing putative virulence functions.

## Materials and Methods

### Genome Sequencing

Sample collection and naming is described in Seth-Smith et al. (2016). Briefly, samples were collected from Greek fish farms during epitheliocystis outbreaks in June 2013 from Argolida or in October 2013 from Arkadia, with names reflecting the year, location, and fish number. Gill arches from individual fish were taken in parallel into 10% buffered formalin, RNALater, and pure ethanol. Gill material and cysts preserved in RNALater or ethanol were micromanipulated using a Leica M165C dissecting microscope. DNA extraction and processing including Genomiphi V2 multiple displacement whole genome amplification (MDA) (GE Life Sciences, Glattbruch, Switzerland) and host DNA depletion NEBNext Microbiome DNA Enrichment Kit (NEB, MA, USA) is described in Seth-Smith et al. (2016) with a summary in table 1. Samples were sequenced on the Illumina MiSeq platform at 6- to 12-plex with 250 bp paired end reads following Nextera library creation. Read data has been submitted to the European Nucleotide Archive (ENA, http://www.ebi.ac.uk/ena/) under study PRJEB7439 with the sample accessions given in supplementary table S1, Supplementary Material online.

**Table 1:** Sample and sequence information

Sample name	# cysts pooled	MDA	Host DNA depletion	% G+C	Total assembly size (Mb)	% Eukaryota*	% Bacteria*	% Unknown*	Ca. Ichthyo. 16S	Genome completeness %	Diversity	Comments
2013Arg12A	12	N	N	41.1	25.1	10.7	3.0	86.3	Y	97.5	1	
2013Arg12B	8	N	N	41.3	88.7	11.1	0.5	88.4	Y	97.5	2	
2013Arg13	10	N	N	37.3	8.9	4.5	12.8	82.8	Y	97.5	4	
2013Arg14A	20	Y	N	38.9	2.7	1.1	35.1	63.8	Y	97.5	1	
2013Arg14B	20	Y	N	45.6	6.4	17.1	0.0	82.9	N	10	1	Excluded, low completeness
2013Arg22	1	Y	N	38.7	2.8	3.2	35.8	61.0	Y	97.5	1	
2013Arg32	1	Y	N	40.4	3.5	9.9	24.5	65.7	Y	87.5	2	Excluded, low completeness, high diversity
2013Arg35	30	N	Y	42.2	15.3	12.6	0.3	87.1	Y	75	2	Excluded, low completeness, high diversity
**2013Arg41**	12	N	N	38.8	3.4	4.4	22.2	73.3	Y	97.5	1	
2013Ark7	gill frags	Y	Y	39.3	9.6	8.0	1.4	90.6	Y	97.5	2	
2013Ark11A	15	N	N	35.4	2.4	0.7	39.5	59.8	Y	97.5	1	
**2013Ark11B**	11	Y	N	37.7	4.1	1.1	30.8	68.1	Y	97.5	2	
2013Ark19	10	Y	N	39.2	3.2	1.1	28.8	70.0	Y	97.5	1	

Metagenome assembly properties, bacterial genome completeness, and diversity. Sample names in bold are those which were annotated and deposited with ENA.

* Assigned by MEGAN

### Assembly of Sequence Data

MiSeq paired-end reads were trimmed and filtered using Trimmomatic (Bolger et al. 2014) to remove adaptor contamination and low-quality regions. Quality controlled reads were assembled using the SPAdes pipeline (Bankevich et al. 2012) with both single cell and multi-cell modes (Nurk et al. 2013). Reads were mapped back to the assemblies and genome assembly likelihoods were computed using Computing Genome Assembly Likelihood [CGAL (Rahman and Pachter, 2013)]. For each sample, the assembly with the higher genome assembly likelihood was retained for downstream analysis (supplementary table S1, Supplementary Material online). *Ca*. Ichthyocystis 16S rRNA gene sequences (EMBL accession numbers: LN612726–LN612730) were searched against each assembly using BLASTN to confirm the presence of targeted pathogens. Hidden Markov models (HMM) of single-copy marker genes (Wu et al. 2013) were searched against each assembly using hmmsearch (Mistry et al. 2013) to evaluate the completeness and diversity of harbored bacterial genomes. Assembled sequences were compared against NCBI nonredundant DNA database (nt) for taxonomic content analysis using MEGAN (Huson et al. 2011).

### Construction of Genome Drafts from Assemblies

For assemblies with estimated bacterial genome diversity of 2 and above, assembled sequences were first classified into different bins using MaxBin (Wu et al. 2014), which groups sequences based on tetranucleotide frequency distribution and coverage distribution using an expectation–maximization algorithm. Individual bins were then checked for the presence of *Ca*. Ichthyocystis 16S rRNA gene sequences. Host contamination was removed from each bin based on either BLAST matches or coverage cutoffs. The diversity of bacterial genomes in each sample was estimated using the 40 single-copy marker genes universal to all sequenced bacteria and archaea (Wu et al. 2013), and taking the rounded average copy number. Completeness of each bin was estimated using these 40 single-copy marker genes and the 107 that are conserved in 95% of all sequenced bacteria (Wu et al. 2013). Two of the mini-metagenomes (2013Arg12B and 2013Arg13) could be seeded with the set of 107 markers; the others were seeded with the set of 40 markers (supplementary table S2, Supplementary Material online). It is known that when seeded with the set of 40 markers, MaxBin tends to split a genome into multiple bins, in which cases bins were combined to represent the final recovered genome draft. Bins were combined only if they were found to harbor complementary single-copy markers and/or *Ca*. Ichthyocystis 16S rRNA gene sequences (supplementary table S2 and fig. S1, Supplementary Material online).

### Construction of Genomic Phylogenetic Trees

Genome phylogenies were built using 30 single-copy marker genes that were found in all genome drafts recovered in this study. Amino acid sequences of the 30 marker genes were extracted from these and downloaded reference genomes, and aligned by ClustalW (Larkin et al. 2007) individually. The alignments were then concatenated and refined using Gblocks (Talavera and Castresana 2007) with default options. MEGA7 (Kumar et al. 2016) was used to build the maximum-likelihood marker gene tree using default settings with 1,000 bootstraps. For comparison, single gene trees were built based on Gblocks edited single gene alignments, with the consensus tree computed using the PHYLIP consensus program (http://evolution.genetics.washington.edu/phylip/phylip.html; last accessed December 2015).

### Whole Genome Alignment and Gene Content Analysis

To confirm the genus boundary, the percentage of conserved proteins (POCP) was computed based on bidirectional BLASTP (Altschul et al. 1990) searches between the two reference genomes for each species as described by Qin et al. (2014). For investigation of species definition, genome drafts were aligned against the two reference representatives using MUMmer (Kurtz et al. 2004) with the default parameter setting, and genome coverage at 95% average nucleotide identity (ANI) was computed. Single nucleotide polymorphisms (SNPs) within MUMmer aligned conserved regions were identified using the same software package. Orthologous genes between the two manually curated reference genomes were identified by bidirectional best BLASTP hit (e-value cutoff of 10^−^^5^). The size of the genus core genome was estimated based on the 942 orthologous gene pairs, representing 0.96 Mb coding sequence. The codons were aligned based on protein alignment using the perl script “pal2nal.pl” (http://www.bork.embl.de/pal2nal/; last accessed December 2015). Orthologous gene pairs were defined using reciprocal best hit based on BLASTP searches. Ratios of nonsynonymous and synonymous substitution rates were computed using KaKs_Calculator (Zhang et al. 2006). All ten implemented methods were initially run with the Ka/Ks ratio distribution found to be tightest for the methods YN (Yang and Nielsen 2000), which was then used with the Hasegawa-Kishino-Yano substitution model (Hasegawa et al. 1985). We went on to investigate genes with Ka/Ks ratio higher than the genome-wide average plus one standard deviation. For visualization of genome alignments using genoplotR (Guy et al. 2010), scaffolds in each genome draft were ordered and oriented into a pseudomolecule based on MUMmer alignment using ABACAS (Assefa et al. 2009).

### Annotation, Kyoto Encyclopedia of Genes and Genomes, and Clusters of Orthologous Groups Analysis

Automated annotation used Prokka (Seeman 2014). For two reference genomes, 2013Ark11B and 2013Arg41, annotation was manually curated in Artemis using BLASTP identities, Pfam, Rfam, SignalP, TMHMM, and Ncoils (http://pfam.xfam.org/, http://rfam.xfam.org/, http://www.cbs.dtu.dk/services/SignalP/, http://www.cbs.dtu.dk/services/TMHMM/and https://launchpad.net/ubuntu/utopic/+package/ncoils; last accessed May 2015). Manual inspection of coding sequences (CDSs), especially for those with low overall identity to known proteins, also used modeling against known structures using SwissModel, partiFold, and Beta-WrapPro for beta-helices and beta-trefoils (http://swissmodel.expasy.org/interactive, http://partifold.csail.mit.edu/TMB/and http://groups.csail.mit.edu/cb/betawrappro; last accessed January 2016) (Arnold et al. 2006). These updated annotated assemblies have been submitted to the ENA (http://www.ebi.ac.uk/ena/) under study PRJEB7439, as have the unordered scaffolds of other genome drafts. Where gap sizes between scaffolds are unknown, an estimated gap length of 2 is given. KEGG2 (Kanehisa and Goto 2000; Kanehisa et al. 2010) analysis was run through KEGG Automatic Annotation Server pathway annotation and mapping to annotate the gene models extracted from each genome draft. The template dataset was customized with 40 beta-proteobacteria species. Bidirectional best BLAST hit was used for the assignment. Pathways were reconstructed and compared using the pathway mapper (Kanehisa et al. 2012). The reference dataset for COG annotation (Galperin et al. 2015) was downloaded from NCBI ftp site (ftp://ftp.ncbi.nih.gov/pub/COG/COG2014/data/; last accessed May 2015). COG annotation was performed using COGcognitor (Kristensen et al. 2010). Comparator genomes used are *Janinthobacterium* sp. Marseille, EMBL accession CP000269 (Audic et al. 2007), and *Chlamydia trachomatis* strain D/UW-3/CX, EMBL accession AE001273 (Stephens et al. 1998).

### Prediction of Secreted Proteins

Translated gene models from each genome draft were screened for the presence of functional type III and IV secretion systems using EffectiveDB (Jehl et al. 2011) and T346Hunter (Martínez-García et al. 2015). Prediction of secreted proteins based on eukaryotic-like domains was performed using EffectiveDB with Z-score refinement.

### Transmission Electron Microscopy

Transmission electron microscopy (TEM) was performed as previously described (Seth-Smith et al. 2016). Samples were fixed in 2.5% glutaraldehyde and 1% OsO_4_ buffered with 0.1 M sodium phosphate (pH 7.4). Samples were dehydrated in an ethanol series, embedded in Epon 812 resin, and ultrathin (90 nm) sections were stained with uranyl acetate and lead citrate. Images were acquired using a Philips CM10.

### Analysis of Gene Families

Multiple alignments of gene families were generated during manual curation of 2013Ark11B and 2013Arg41 gene models. HMMs were built and searched against scaffolds from genome drafts using hmmsearch. Gene families (IchFam1-25) are defined in the annotation under/note qualifier. Phylogenies of families in the two reference genomes were generated from amino acid or nucleotide alignments (clustalo and PhyML within Seaview) (Gouy et al. 2010). The phylogeny of IchFam18 from all genome drafts was generated from nucleotide alignments using MEGA7.

## Results and Discussion

### Sequencing of Cyst Samples and Assembly of Mini-Metagenomes

Microdissected infected gill filaments and separated epitheliocysts, single or in pools, were processed for sequencing (table 1). Thirteen samples were sequenced, and the representative mini-metagenomes were assembled using SPAdes. The assemblies were heterogeneous in terms of genome metrics including N50 and total assembled length (supplementary table S1, Supplementary Material online). The presence of *Ca*. Ichthyocystis in each assembly was first confirmed by mapping against the representative 16S rRNA gene sequences. Assembled scaffolds were then compared with the NCBI nucleotide database (nt) for estimation of species composition. To determine whether individual *Ca*. Ichthyocystis genome drafts of good quality could be recovered, the assemblies were evaluated using the single-copy marker genes universal to all sequenced bacteria and archaea, and the diversity and completeness of bacterial genomes were estimated (table 1).

The majority of the assembled scaffolds have no significant matches to known sequences. Contamination from eukaryotic sequences was found in all assemblies, with identifiable sequences comprising up to 10% of the assembly. All assemblies with the exception of 2013Arg14B showed hits to the *Ca*. Ichthyocystis 16S rRNA gene sequences. This assembly also lacks 90% of the bacterial marker genes, and sequence analysis suggested most of the known sequences are of eukaryotic origin. Assemblies 2013Arg32 and 2013Arg35 were each found to contain two different but incomplete bacterial genomes. These three samples were excluded from further analysis. The remaining ten assemblies had the potential to construct *Ca*. Ichthyocystis genome drafts. Estimated by the copy number of marker genes, each assembly contains between one and four different bacterial genomes (table 1).

### Construction of *Ca*. Ichthyocystis Genome Drafts

For the six mini-metagenome assemblies estimated to contain only one close-to-complete bacterial genome, the corresponding genome draft was defined by scaffolds above 1 Kb and above a coverage threshold derived for each assembly, with the aim of removing sequences of eukaryotic origins (supplementary fig. S2, Supplementary Material online). For the four mini-metagenome assemblies’ harboring more than one bacterial genome, sequences were classified by combining multiple evidences. First, the sequences were categorized using MaxBin, which binned sequences using an expectation–maximization algorithm based on both tetranucleotide frequencies and scaffold coverage (supplementary table S2 and fig. S1, Supplementary Material online). The sequence bins were then checked for sequence similarity to known sequences, and eukaryotic sequences were further removed from each bin by sequence similarity or extra coverage thresholds. Finally, the completeness of scaffold bins were also evaluated using the set of bacterial marker genes. Scaffold bins from assembly 2013Ark7 harbored too few bacterial marker genes (supplementary table S2, Supplementary Material online) and the scaffold coverage was too low to effectively separate the bacterial and eukaryotic scaffolds (supplementary fig. S1, Supplementary Material online) to continue with the analysis. This sample was derived from gill filaments, showing that the microdissection of cysts prior to sequencing improves the likelihood of successful downstream analysis, especially when dealing with low sequencing depth.

**Fig. 1.—:**
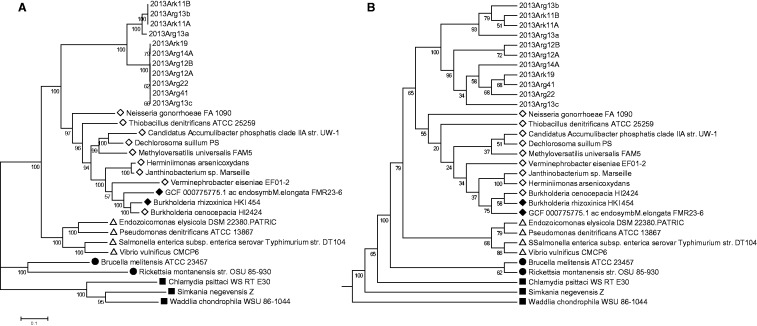
Phylogeny of *Ca*. Ichthyocystis. (*A*) Maximum-likelihood tree based on the concatenated protein sequences of 30 marker genes extracted from *Ca*. Ichthyocystis genome drafts, with representative bacteria species from gamma-proteobacteria (triangles), alpha-proteobacteria (circles), and the Chlamydiae (squares) included as outgroups. Filled shapes represent bacteria with obligate intracellular lifestyles. The root was arbitrarily placed at the Chlamydial lineage. Epitheliocystis pathogens sequenced in this study form two major clades within the beta-proteobacterial lineage (diamonds). In total 3695 sites were used, which were extracted from the 5767 sites in the original protein alignment by Gblocks after eliminating poorly aligned and divergent regions. The scale bar indicates the number of substitutions per site. (*B*) Consensus tree of the 30 single marker gene trees. The numbers shown next to the branches indicates the percentage of times the same grouping occurs in single gene trees. For each marker gene, poorly aligned and divergent regions were excluded using Gblocks. Individual maximum-likelihood trees were constructed in MEGA7. All 30 single gene trees were then analyzed using PHYLIP consensus to generate the consensus tree. The grouping of the 11 genome drafts at genus and species level are highly similar in (*A*) and (*B*), with high support values, suggesting that these draft genomes represent single genotypes.

From the nine remaining assemblies, we constructed 11 genome drafts, with three originating from a single sample (2013Arg13) (table 2). To evaluate the accuracy of the genome drafts, and confirm the phylogenetic context of these novel bacteria, we constructed a tree of 30 concatenated single-copy marker genes identified within these genome drafts and reference genomes from major bacterial lineages (fig. 1). This phylogeny shows the same topology as the phylogenetic classification of the genus using 16S rRNA gene sequences (Seth-Smith et al. 2016). All 11 genome drafts fall into one novel lineage within the beta-proteobacteria, with the four *Ca*. I. hellenicum-like genomes and seven *Ca*. I. sparus genomes forming two distinct clades. The POCP analysis confirmed that all genomes belong to the same genus (cutoff 50%) (Qin et al. 2014). Based on the species cutoff of whole genome alignment [over 69% genome coverage with ANI of 95% (Goris et al. 2007)], all genome drafts but one belongs to one of the two described species. Draft 2013Arg13a aligned against the *Ca*. I. hellenicum reference genome 2013Ark11B has less than 1% genome coverage at 95% ANI and therefore likely represents a new species of *Ca*. Ichthyocystis that is closer to *Ca*. I. hellenicum than *Ca*. I. sparus, which is also reflected in the marker gene phylogeny (fig. 1).

**Table 2:** **Characteristics and gene content of constructed *Ca*. Ichthyocystis genome drafts.**

Genome draft	2013Arg12A	2013Arg12B	2013Arg13a	2013Arg13b	2013Arg13c	2013Arg14A	2013Arg22	2013Arg41	2013Ark11A	2013Ark11B	2013Ark19
# scaffolds	244	703	80	76	535	100	110	208	111	145	203
Total length (bp)	2299389	2603008	1329696	1500386	2024494	2386839	2443755	2589532	2248688	2363907	2457116
Largest scaffold (bp)	247651	157533	131237	111159	37780	294370	280014	254483	160105	271851	374916
N50	59601	7483	43403	58780	5739	107277	154231	60724	60216	140069	81911
% G+C	38.2	38.33	40	37.77	39.2	38.49	38.34	38.33	35.37	35.14	38.23
Completeness, 40 bacterial and archaeal markers	97.5	97.5	97.5	97.5	95	97.5	97.5	97.5	97.5	97.5	97.5
Completeness, 107 bacterial markers	93.46	93.46	89.72	86.92	90.65	93.46	93.46	93.46	94.39	94.39	93.46
Completeness, 311 betaproteobacterial markers	81.99	78.15	74.28	73.95	79.42	81.99	81.99	81.99	81.35	81.35	82
# predicted genes*	1695	2004	1178	1208	1616	1678	1699	1818	1639	1714	1759
# KEGG annotated genes (%)	658 (38.8)	669 (33.4)	613 (52.0)	613 (50.7)	644 (40.0)	658 (39.2)	661 (38.9)	665 (36.5)	665 (40.6)	668 (38.64)	658 (37.4)
# COG annotated genes (%)	965 (56.9)	1068 (53.3)	859 (72.9)	849 (70.3)	971 (60.1)	971 (57.9)	959 (56.4)	982 (53.9)	1007 (61.4)	1012 (58.53)	962 (54.7)
Assigned species (*Ca.* I.)	sparus	sparus	(hellenicum)^**^	hellenicum	sparus	sparus	sparus	sparus	hellenicum	hellenicum	sparus

* Gene models were predicted using prokka without manual annotation, except for the reference genomes 2013Ark11B and 2013Arg41, and may thus be underestimations given the novelty of many of the genes and their partial nature on the multiple scaffolds.

** This sample may constitute a third species, but is more closely related to *Ca*. I. hellenicum than *Ca*. I. sparus

We previously reported sequence diversity within species based on mapping of sequencing reads to the genes encoding conserved proteins in the two reference genomes (Seth-Smith et al. 2016). Multiple genome comparisons within each species further revealed that the gene content diversity among *Ca*. I. hellenicum drafts is higher than that among *Ca*. I. sparus drafts. Within species *Ca*. I. hellenicum, excluding 2013Arg13a, the POCP ranges from 70% to 98%, while among all the seven *Ca*. I. sparus drafts the POCP is above 90%. In both species, the SNP density in 95% ANI conserved regions averages at 1–2 SNPs per Kb.

### General Genome Features

Eight of the 11 genome drafts are close-to-complete (completeness > 93.5%, table 2), as estimated using the 107 marker genes universal to most bacteria (Wu et al. 2013), and range in size from 2.3 to 2.6 Mb made up of 100–703 scaffolds. These statistics encourage us that our genome drafts are as complete as possible, given the difficulties associated with our starting material, and can be considered to be high-quality drafts (Chain et al. 2009). The three genomes constructed from the single mini-metagenome sample 2013Arg13 (10 pooled cysts) are less complete (87–91% completeness), but represent at least two *Ca*. Ichthyocystis species, indicating multiple infections by different pathogen species in a single host. The G + C content of the *Ca*. I. sparus genomes is approximately 38%; that of *Ca*. I. hellenicum is approximately 35% (table 2). The reduced genome size compared with other beta-proteobacteria (Seth-Smith et al. 2016) and low G + C content is characteristic of genomes of symbiotic bacteria (Moran 2003), thought to be consequences of small effective population sizes and limited opportunity for genetic exchange (McCutcheon and Moran 2012).

**Fig. 2.—:**
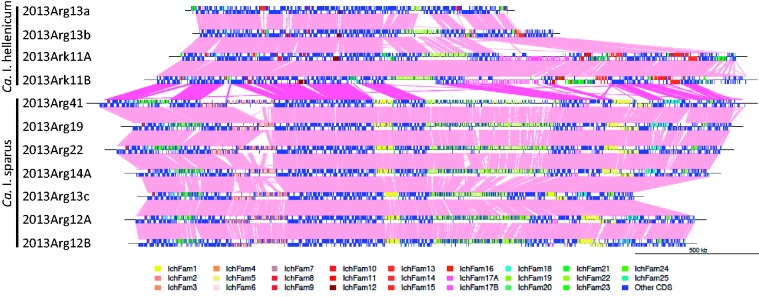
Global pairwise comparisons of *Ca*. Ichthyocystis genome drafts. Scaffolds from each genome draft were ordered against the relevant reference draft using ABACAS, and visualized with the orthologous matches using genoPlotR. Each draft is shown as a line, with tick marks above and below the sequence lines representing the predicted CDSs on the plus strand and the minus strands. Gene family members are marked with family specific colors as shown in the key. Direct orthologous matches are shown by lines connecting genome drafts, with interspecies matches highlighted in hot pink, and intraspecies in light pink. The draft 2013Arg13a may represent a novel species, but is more closely related to *Ca*. I. hellenicum.

Gene prediction and automatic annotation was performed on each genome draft using Prokka (fig. 2). Reference draft genomes from the two main species (2013Ark11B for *Ca*. I. hellenicum and 2013Arg41 for *Ca*. I. sparus) were further manually annotated, and the updated versions have been deposited with EMBL. For these, scaffold rearrangement was performed under the assumption that the genomes are syntenic. A “core” genome of the genus is apparent when comparing these two reference genomes (fig. 2), which comprises approximately 1.0 Mb coding sequence. These reference genomes build on the previous draft assemblies reported (Seth-Smith et al. 2016) and demonstrate that we can obtain almost complete draft genomes from preserved epitheliocystis infected material, resulting in annotation-directed improvement standard drafts (Chain et al. 2009).

The features of the two reference genome drafts are given in table 3. The relatively small size of the *Ca*. Ichthyocystis genomes, and in particular the core, provides some initial evidence of a compact genome which has undergone genome reduction. The very low number of pseudogenes within the core genome is an indicator that the core is stable. This, in addition to the high similarity in core gene content and arrangement (fig. 2), suggests that the functions encoded within the core genome have not changed greatly since the most recent common ancestor (MRCA) of the genus, despite nucleotide variation of approximately 70% between the core of the two reference drafts. The drafts possess fewer regulators than would be expected in a free-living organism, a phenomenon which is also indicative of intracellular adaptation (Merhej et al. 2009). No evidence of any mobile elements exists, with the exception of a CDS, predicted to encode a XerC family Phage integrase, present in both genomes (*Ark11_1163* corresponding to *Arg41_1271*, approximately 40% shared amino acid identity), but not in a core location.

**Table 3:** **Features of reference *Ca*. Ichthyocystis genome drafts.**

Genome draft	2013Ark11B	2013Arg41
Genome draft size (bp)	2364195	2589311
# scaffolds	145	208
% G+C	35.17	38.45
# CDSs	1714	1818
Coding percentage	80.1	74.4
Mean size of CDSs (bp)	1102	1073
%G+C in CDSs	36.55	40.06
# tRNA	39	37
# rRNA operons	2	2
# pseudogenes	0	30*
# regulators	41	38
# gene family CDSs	481 (28%)	534 (29%)

Data based on manually annotated draft genomes of *Ca*. I. hellenicum (2013Ark11B) and *Ca*. I. sparus (2013Arg41).

* Of these, 28 are duplicated versions of genes with intact versions elsewhere in the genome: 13 are enolase pseudogenes (intact at *Arg41_0208*), five are thiodoxin reductase pseudogenes (intact at *Arg41_1126*), two are 23S rRNA methyltransferase pseudogenes (intact at *Arg41_1114*), and eight are putative toxin pseudogenes; all hypothesized to have resulted through gene family amplification mechanism duplication and subsequent pseudogenization. One regulator pseudogene has no equivalent ortholog in 2013Ark11B, and the only possible loss of core function in the genome draft 2013Arg41 compared with 2013Ark11B is that of the translation initiation factor IF-3 (*Arg41_1259*), for which no suitable start codon was identified

Analysis of CDSs potentially under positive selection in the two species as represented by genome drafts 2013Ark11B and 2013Arg41 identified 104 gene pairs with Ka/Ks ratios above the genome-wide average (average plus one standard deviation among all 942 gene pairs). Of these, the vast majority are function unknown (supplementary table S3, Supplementary Material online) and many are putative membrane proteins. This perhaps reflects the selective pressures on external structures, although it should also be borne in mind that perhaps it is the accessory genes, which often do not occur as orthologs and therefore were outside the reach of this method, which are those most likely to be under selection from the host and other pressures. Of the gene families shared between the two genome drafts, 15 members were identified as putative orthologous pairs, of which ten were found to be under positive selection (supplementary table S3, Supplementary Material online).

### Metabolic Pathway Analysis

To reconstruct metabolic pathways, we annotated all genome drafts using KEGG Ontology and evaluated the functional units as defined by the KEGG modules. The profiles of the KEGG modules present are highly similar in all drafts, especially in the eight close-to-complete genome drafts. These have 35 complete functional units: 19 representing metabolic pathways, 15 forming molecular machineries, and one essential functional set (aminoacyl-tRNA synthases) (supplementary table S4, Supplementary Material online). That all genome drafts give very similar profiles further encourage us that our drafts are close-to-complete.

The machinery for genetic information processing, including DNA polymerase III complex, RNA polymerase, and ribosomal proteins, is complete in most drafts. All major bacterial ATP synthesis machineries are present, as are six complete transporter systems: the ABC-2 type transport system and phosphate transport system (both ubiquitous in over 80% of the bacterial genomes annotated in the KEGG genome database), lipoprotein-releasing system, glutamate/aspartate transporter, branched-chain amino acid transporter and phospholipid transporter, the latter four of which are not so widely spread in bacteria and are therefore more likely to be niche-specific.

Complete pathway modules were found in carbohydrate and lipid metabolism (11 functional units), as well as nucleotide, cofactor, and vitamin metabolism (eight functional units), indicating that these are functional pathways in *Ca*. Ichthyocystis. However, all functional units related to amino acid metabolism are either totally absent (histidine, arginine, and proline) or incomplete (all other amino acids) (supplementary fig. S3, Supplementary Material online). The same was observed in all corresponding mini-metagenomes, showing that the lack of these pathways is not due to the process of genome draft construction (supplementary table S5, Supplementary Material online). Given that all essential molecular machinery, the full set of bacterial aminoacyl-tRNA synthases, and other essential pathways are found complete in the same drafts, we believe that the lack of capacity for amino acid metabolism in these bacteria is strong evidence of their obligatory intracellular lifestyle. We hypothesize that they scavenge amino acids from hosts using the amino acid transport systems identified within the genomes: glutamate/aspartate transporter and branched-chain amino acid transporter.

Development of an intracellular lifestyle is usually accompanied by gene loss (Toft and Andersson 2010), exemplified by *Mycobacterium leprae* (Cole et al. 2001). This process occurs first through pseudogenization, then progressive deletion of the nonfunctional genes. The genomes of *Ca*. Ichthyocystis seem consistent in their metabolic core, indicating that the process of functional gene loss occurred in the distant history of the genus, prespeciation, and that further reduction is not ongoing. This stability of the core genome may also indicate that there is a host restriction on these pathogens.

### Functional Analysis of COG

As the KEGG annotation only assigned the function to approximately 30–40% of annotated CDSs, we analyzed all genomes with COG families to gain further functional insights. In this way, approximately 50–60% of predicted CDSs could be annotated (table 2), with gene numbers for individual functional categories again similar among all 11 genome drafts (supplementary fig. S4, Supplementary Material online). Comparing these results to the COG compositions of the most closely related free-living beta-proteobacterium with an available genome sequence, *Janinthobacterium* sp. Marseille (4.1 Mb) (Audic et al. 2007), and the less closely related obligate intracellular pathogen *Chlamydia trachomatis* (1 Mb), the COG composition profile (and genome size) of *Ca*. Ichthyocystis is found to be more similar to that of *C. trachomatis* (supplementary fig. S5, Supplementary Material online).

The most abundant functional category in both *Ca*. Ichthyocystis and *C. trachomatis* relates to translation, ribosomal structures, and biogenesis (J), a category found to be the only functional group that was not affected by genome reduction in bacteriocyte endosymbiont genomes comparing to free-living ones (Zientz et al. 2004). It is interesting to speculate whether this influences the replication rate of the bacteria, many of which were seen to be in a state of active division in cysts by electron microscopy (EM) (Seth-Smith et al. 2016). Six functional categories have different presences in *Ca*. Ichthyocystis when comparing to *Janinthobacterium* (*P* value < 0.001 by Fisher’s exact test) but not when compared to *C. trachomatis*. Among them, three were enriched: cell cycle control cell division and chromosome partition (D), nucleotide transport and metabolism (F) and coenzyme transport and metabolism (H); the other three were depleted: transcription (K), inorganic ion transport and metabolism (P), and function unknown (S). The functional category of extracellular structures (W) is significantly enriched in *Ca*. Ichthyocystis compared with both these reference species (*P* < 0.05, Fisher’s exact test), and includes genes encoding proteins involved in the type IV pilus (Tfp, see below, supplementarytable S6, Supplementary Material online).

### Secretion Systems in *Ca*. Ichthyocystis

**Fig. 3.—:**
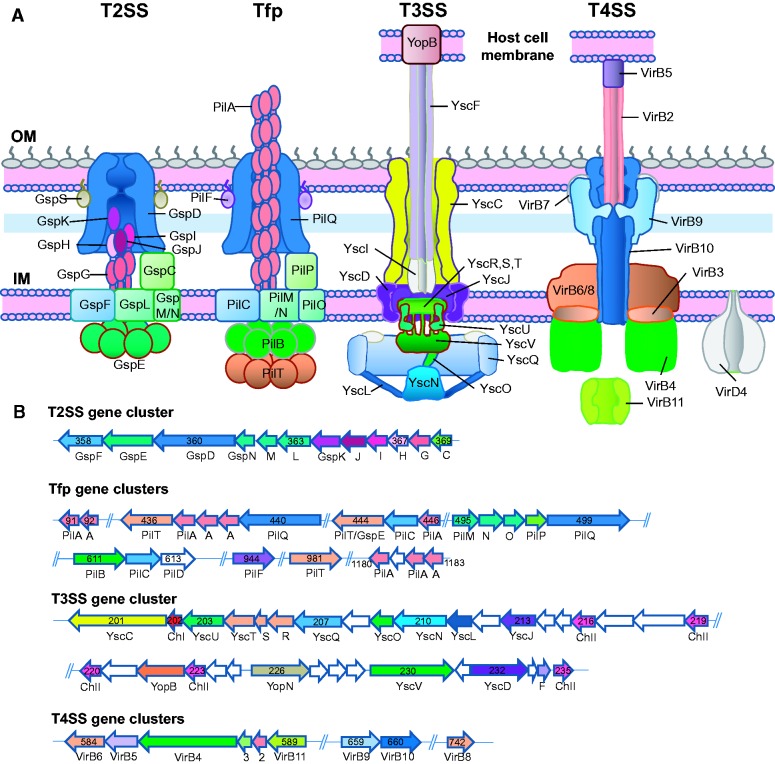
Surface structure and secretion systems identified in *Ca*. Ichthyocystis, and CDSs involved in their production. (*A*) Schematic of structures identified: T2SS, Tfp, T3SS, and T4SS, with the peptidoglycan layer depicted as a light blue band and the absent lipopolysaccharides depicted in gray. Nearly all components of the four secretion systems have been located within the reference genomes of both species, with only the GspS pilotin of the T2SS, YscI of the T3SS and VirB7 and VirD4 of the T4SS being as yet unidentified. OM, bacterial outer membrane; IM, bacterial innner membrane. (*B*) Gene clusters identified as being involved in the structural components of, respectively, T2SS, Tfp, T3SS, and T4SS. The colors in each macromolecular structure in (*A*) are reflected in the corresponding CDSs, which are numbered relative to the genome draft of 2013Ark11B (*Ca*. I. hellenicum, locus tag Ark11_0) but are equivalent in 2013Arg41 as described in the text. CDSs within the clusters with as yet unassigned functions are shown in white. A single class I T3SS chaperone is labeled with ChI (red) along with a family of five class II (ChII) T3SS chaperones (brick red). The schematic models shown for the T2SS and Tfp are based on the figure from Korotkov et al. (2012), the model for the T3SS from Diepold and Armitage (2015, figure 1) and the T4SS of Costa et al. (2015, figure 1a).

The successful invasion of host cells depends on the ability of the pathogen to interact with and adhere to its host. Such interaction is also mediated by delivering effectors such as virulence factors and toxins into host cells to influence the host response. Gram-negative pathogens are known to use secretion systems to transport proteins and help host invasion (Costa et al. 2015). For *Ca*. Ichthyocystis, we have microscopically observed the impact of the infection on the host: forming a controlled environment within the bacteria-containing compartment for replication, and causing changes in the epithelial cell environment to form the interdigitating processes seen under EM (Seth-Smith et al. 2016). Components of type II, III, and IV secretion systems (T2SS, T3SS, T4SS; fig. 3) were detected in the *Ca*. Ichthyocystis genome drafts by both KEGG (supplementarytable S4, Supplementary Material online) and COG (supplementary table S6, Supplementary Material online) analysis, with a correspondingly large collection of predicted T3SS and T4SS effectors.

Manual annotation of the reference draft genomes of 2013Ark11B and 2013Arg41 identified a cluster of 12 CDSs encoding components of a putative T2SS operon (*Ark11_0358-0369/Arg41_0522-0533*; fig. 3) (Korotkov et al. 2012). This cluster includes the gene encoding an outer membrane secretin (GspD) as well as components of the inner membrane platform (GspFLM) and the protein linking the inner membrane platform with the secretin (GspC). The putative pseudopilins (GspHIJK) which cap a growing pilus and the major pseudopilin (GspG) are also encoded by CDSs in this cluster, as is the secretion ATPase (GspE).

All *Ca*. Ichthyocystis genome drafts contain a large gene cluster encoding the proteins which make up a nonflagellar type T3SS or injectisome (*Ark11_0201-0235/Arg41_0282-0318*; fig. 3). Identified CDSs include those encoding five proteins which make up an inner membrane export apparatus (YscRSTUV) which is surrounded by the inner (YscJ) and outer (YscD) ring proteins which connect with the secretin ring (YscC) of the outer membrane, which encloses the needle made up of multiple YscF subunits arranged in a helix with 5–6 subunits per turn. We have not yet been able to identify a gene encoding the inner rod protein YscI, which is thought to anchor the needle to the inner membrane export complex. The cytosolic ATPase (YscN) and associated proteins (YscQL) are also present. The pore forming hydrophobic translocator YopB could also be identified; thus, almost all components of the T3SS are represented. One T3SS type I chaperone (Ark11_0202/Arg41_0283) and a family of five type II chaperones (Ark11_0216, 0219, 0220, 0223, 0235/Arg41_0297, 0302, 0303, 0306, 0308) are encoded within the operon. Most of the extracellular components of the T3SS cannot be identified through sequence homologies, but several CDSs encoding hypothetical proteins are found in key locations in the operon and are likely to assume these roles. Due to this large operon, conserved between the species, and the large number of predicted effector proteins in all drafts, we assume this T3SS to be functional.

Most *Ca*. Ichthyocystis genome drafts were predicted to have a functional T4SS, not located in single gene clusters, but distributed across several scaffolds (*Ark11_0584-0589, 0659-0660, 0742/Arg41_0739-0744, 0815-0816, 0919*; fig. 3). The T4SS appears to belong to the P-type, which are generally made up of 12 proteins: VirB1–VirB11 plus VirD4, but which is variable across bacteria species (Juhas et al. 2008). Within our draft genomes, we have identified the major core components (VirB2–6, 8–11) with only the VirB1, 7, and VirD4 currently unannotated. All genome drafts were found to have a repertoire of genes encoding predicted T4SS effectors (see below). This indicates that the *Ca*. Ichthyocystis P-T4SS could well extend a pilus associated with the secretion channel, and substrates may be directly secreted into the host cell through the outer membrane pore.

### Cell Wall and Extracellular Structures

Changes in the outer structures of a bacterial cell often occur during evolution from a free living to an intracellular lifestyle (Toft and Andersson 2010). The peptidoglycan biosynthesis pathway encoded within the *Ca*. Ichthyocystis genome drafts is largely complete, and more similar to the pathway found in the related free-living bacterium *Janinthobacterium* sp. Marseille than to that in *C. trachomatis*. The relevant CDSs are found in several gene clusters, located with other CDSs involved in cell division and phospholipid transport (*Ark11_0165-0174, 0401-0408, 0629-0632, 1381-1383/Arg41_0246-0255, 0575-0582, 0786-0789, 1551-1553*).

Lipopolysaccharide (LPS) is often found in the outer membrane of Gram-negative bacteria, with strong immunogenic properties. LPS biosynthesis and export systems are absent from our genome drafts, with CDSs predicted to encode only one or two enzymes present: D-sedoheptulose 7-phosphate isomerase (*Ark11_1179*/not present in 2013Arg41) and UDP-2,3-diacylglucosamine hydrolase (*Ark11_0696/Arg41_0851*). In contrast, LPS export systems are complete in *C. trachomatis* and *Janthinobacterium*, with both able to synthesize Lauroyl-KDO2-lipid IV4, which is the final intermediate for the synthesis of Kdo2-Lipid A, a truncated LPS substructure with endotoxin activity similar to native LPS (Raetz et al. 2006). The *Janthinobacterium* reference genome harbors two complete enzymatic processes involved in this pathway, consisting of 13 enzymes, with nine in the reaction starting with UDP-N-acetyl-D-glucosamine, and four in the reaction utilizing D-ribulose-5-phosphate. The *C. trachomatis* reference genome also contains the two different processes and 11 of the 13 enzymes, in which the process from UDP-N-acetyl-D-glucosamine to Lauroyl-KDO2-lipid IV4 is complete. Lack of LPS has been reported before in other bacteria, where alternative structures may exist (Takayama et al. 1987; Kawahara et al. 1991; Vinogradov et al. 2001; Keck et al. 2011), and also in the obligate intracellular bacterium *Orientia tsutsugamushi* (Nakayama et al. 2008), where its absence may help to avoid the host innate immune system in the initial phase of infection.

Tfp are unique appendages on the bacterial surface and have been found in diverse Gram-negative bacteria. They play important roles in many cellular processes, such as cell movement, bacterial adherence, host tissue invasion, and other pathogenesis-related events (Shi and Sun 2002). For example, the pathogenic Neisseriae (*Neisseria meningitidis* and *N. gonorrhoeae*) initiate colonization of mucosal epithelia using Tfp (Higashi et al. 2009; Coureuil et al. 2012; Eriksson et al. 2012). Although closely related to, and evolutionarily derived from the T2SS, we have attempted to identify the CDSs encoding the Tfp in *Ca*. Ichthyocystis. In contrast to the T2SS, whose genes are encoded together in a single cluster, as is typically the case (Korotkov et al. 2012), genes encoding the Tfp are spread over five clusters. The CDS encoding PilQ, the outer membrane Tfp secretin, is in the same cluster as those encoding members of the inner membrane platform, PilMNOP (*Ark11_0495-0499/Arg41_0659-0663*; fig. 3). Elsewhere in the genome is another cluster (*Ark11_0436-0446/Arg41_0600-0610*) including CDSs encoding an alternative inner platform gene PilC, two versions of PilT, and an additional PilQ. A putative prepilin peptidase (PilD; Ark11_0613/Arg41_0770) is encoded in a cluster with *pilB* and *pilC* (*Ark11_0611-0612/Arg41_0768-0769*) which, based on homologies, have been assigned to the Tfp rather than the evolutionarily related T2SS. A CDS encoding PilF, a Tfp assembly protein, is found separately (*Ark11_0944/Arg41_1165*), as is a third PilT (*Ark11_0981/*not present in Arg41). That there are up to three identified genes encoding variants of the ATPase PilT, which provides the driving force for pilus retraction, indicates that *Ca.* Ichthyocystis is likely to be motile at some stage in its lifecycle. Up to nine putative pilin encoding CDSs have been identified within this operon (*Ark11_0437-0439, 0446/Arg41_0601-0603, 0610*) and elsewhere in the genomes (*Ark11_0091-0092, 1180, 1182-1183/Arg41_0098-0099, 1251-1253*, the latter cluster being variable as compared with the equivalent in 2013Ark11B), and might provide alternative functions, as may the putative minor Tfp pilins PilE and PimT (Ark11_0661-0662/Arg41_0817-0818), which curiously are encoded adjacent to the T4SS CDSs encoding VirB9 and VirB10, indicating perhaps a coordinated regulation of certain elements of these two systems. This large repertoire of components may reflect alternative functions for different stages of the infection. It should be noted, however, that given the close homologies between the T2SS and the Tfp, a definitive assignment would require experimental verification. The roles of Tfp in *Ca*. Ichthyocystis cell motility and pathogenesis would be interesting topics for future studies.

In the genome drafts 2013Ark11B and 2013Arg41, genes encoding for eukaryotic-like proteins (ELPs) were predicted and checked against manual annotation. ELPs are thought to occur at higher frequency in genomes of host-associated bacteria compared with nonhost-associated bacteria, indicating that they might be used to mediate the host behavior. Within this category, several CDSs containing tetratricopeptide repeats were found in both genomes (*Ark11_0216, 0219-0220, 0223, 0463, 0948/Arg41_0297, 0302-0303, 0306, 0627, 1169*) and may play roles as class II chaperones in the T3SS, and in Tfp biosynthesis (Cerveny et al. 2013).

The beta-barrel outer membrane proteins (OMPs) fulfill a number of essential functions in pathogenic Gram-negative bacteria, including adhesion, signaling, and transport of nutrients and ions. A precondition for the presence of OMPs or porins is an operative outer membrane beta-barrel assembly machinery complex (Bakelar et al. 2016), which we have identified within the genome drafts of *Ca*. Ichthyocystis. This comprises the central component BamA (Ark11_0113/Arg41_0110), itself containing a C-terminal 16-strand beta-barrel localized in the outer membrane, which forms a complex with the lipoproteins BamB (Ark11_0949/Arg41_1170), BamC (Ark11_1189/Arg41_1375), and BamD (Ark11_1072/Arg41_1278), stabilized by binding of the lipoprotein BamE (Ark11_1236/Arg41_1441). BamB is thought to function as a scaffold for acceptance of nascent OMPs being delivered from the inner membrane Sec-translocon by the chaperone and proline-isomerase SurA (Ark11_0703/Arg41_0857).

Multiple porins could be identified, however, no families were found, as has been shown for OmpA in *Waddlia chondrophila* (Bertelli et al. 2010) or for the chlamydial type V secretion system autotransporters (Vasilevsky et al. 2016). Curiously, a CDS putatively encoding OmpF porin (*Ark11_0464/Arg41_0628*) was found next to the *tol-pal* operon (*Ark11_0458-0463/Arg41_0622-0627*), products of which forms part of the cell division complex, essential for outer membrane integrity, but which together with OmpF can be hijacked to form a colicin-toxin translocation machine. A putative colicin V production gene (*Ark11_0967/Arg41_1188*) is found within the *purF* operon. Other porins include a predicted eight-strand OmpA-like protein (Ark11_0039/Arg41_0048), a porin with closest homologies to the outer membrane anion selective 16-strand Omp32 (Ark11_0647/Arg41_0804) as well as a 14-strand outer membrane long-chain fatty acid transporter, FadL (Ark11_0576/Arg41_0732).

**Fig. 4.—:**
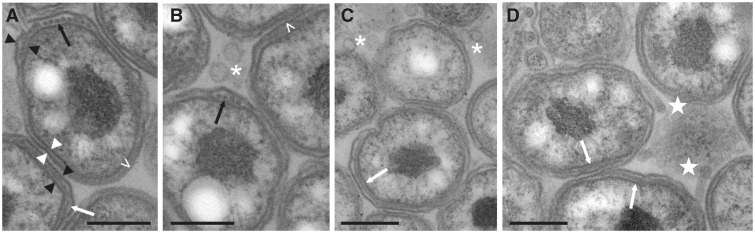
TEM of *Ca*. Ichthyocystis bacteria. The bacterial double membranes, separated by 14–17 nm (*A*, white triangles) can be clearly seen in all images. Between these, a row of small electron dense particles (*A* and *B*, black arrows) or a filament (*A*, *C* and *D*, white arrows), can often be seen, in which case the inner and outer membranes are separated by 30–35 nm (*A*, black triangles). These structures may represent periplasmic filaments cut cross-sectionally or longitudinally. On the opposite pole from these, the double membrane is less distinct and often appears to be bridged by a series of fine structures (*A*, <) which, in some instances, also appear to connect closely opposing bacteria (*B*, <). Vesicles in between bacteria are commonly observed (*B*, *C*, *). (*D*) Structures projecting from the bacteria (white stars). Scale bars: (*A*) and (*B*): 0.2 μm; (*C*) and (D): 0.3 μm.

Using TEM of infected tissue, we visualized several intriguing surface structures from *Ca*. Ichthyocystis bacteria (fig. 4). In many bacteria, series of periplasmic structures can be seen, which may be filaments running between the bacterial surface membranes observed in either cross- or longitudinal- section, and projections from the bacteria are also visible. How these relate to the secretion systems and structures discussed above will be fascinating to investigate in more detail. Multiple extracellular vesicles are commonly seen in between the bacteria within the epitheliocystis infectious lesions (fig. 4), which may be outer membrane vesicles, thought to have roles in pathogenesis and intercellular interactions, and as such represents a further export system of Gram-negative bacteria (Roier et al. 2016). Implicated in the biogenesis is the VacJ/MlaB ABC (ATP-binding cassette) transport system or phospholipid transporter, thought to be important for maintaining the lipid asymmetry between the inner and outer membranes and encoded by a conserved cluster of genes in most Gram-negative bacteria (*Ark11_0169-0174/Arg41_0250-0255*).

### Gene Families

A major characteristic of the genomes of *Ca*. Ichthyocystis is the presence of a large number of gene families with varying size and species distribution. The gene families have been manually cataloged within the assembled reference genome drafts of 2013Ark11B and 2013Arg41. We have defined 26 families in total, named IchFam1-25 (see qualifier/note in the annotation), and screened these across all the ordered genomes using hmmsearch (supplementary table S7, Supplementary Material online). IchFam 8-15, 17A, and 23 are present only in genomes of *Ca*. I. hellenicum; IchFam1, 2, 4–7, 24, and 25 are present only in *Ca*. I. sparus genomes; and IchFam3, 16, 17B, 18–22, and 24 have members in both species. Three of the families have over 100 members in the reference genomes, and the total number of CDSs involved identified to date is 481 in the genome of 2013Ark11B and 534 in 2013Arg41, although there are likely to be additional families and members not yet identified. A summary of the features of these gene families and their members is given in table 4. Such extensive gene duplication is rarely seen within the genomes of intracellular bacteria (Gevers et al. 2004), which usually have compact genomes.

**Table 4:** Numbers, distribution, and characteristics of gene family members within the manually annotated reference genome drafts 2013Ark11B and 2013Arg41

Family name	**2013Ark11B**			**2013Arg41**			Predicted # arrays	Strand switch	Gene product predicted function	Comments
	**#**	**# predicted T3 effectors**	**# predicted T4 effectors**	**#**	**# predicted T3 effectors**	**# predicted T4 effectors**				
IchFam1	0			84	58	3	2	Y	Putative toxin/hypothetical	
IchFam2	0			101	50	12	1	Y	Coiled coil/hyp/exported	
IchFam3	0			10	1	0	1	Y	Putative ATPase/putative membrane	
IchFam4	0			2	2	0	1	N	Putative membrane	Opposite IchFam10 in genome
IchFam5	0			3	3	0	1	N	Coiled coil/hypothetical	
IchFam6	0			4	3	0	1	N	Coiled coil/hypothetical	
IchFam7	0			5	3	1	1	N	Hypothetical	At a rearranged genomic location
IchFam8	6	5	2	0			1	N	Coiled coil/hypothetical	Within IchFam18 array
IchFam9	8	3	0	0			1	N	Hypothetical	
IchFam10	21	9	1	0			1	Y	Putative membrane	Opposite IchFam4 in genome
IchFam11	33	14	0	0			1	Y	Coiled coil/hypothetical	Mixed with IchFam23 array
IchFam12	23	4	0	0			1	N	Putative membrane	
IchFam13	27	20	3	0			1	Y	Coiled coil/hypothetical	
IchFam14	36	16	8	0			2	Y	Coiled coil/hypothetical	
IchFam15	30	16	4	0			1	N	Coiled coil/hypothetical	At a rearranged genomic location
IchFam16	1	0	0	3	0	0	1	N	Putative membrane/hyp/exported	Clear *in situ* expansion
IchFam17A	65	43	1	0			1	N	Coiled coil/hypothetical	
IchFam17B	21	20	2	8	5	2	1	N	Coiled coil/hypothetical	
IchFam18	5	4	1	3	2	2	1	N	Coiled coil/hypothetical	Mixed with IchFam8 array
IchFam19	7	0	1	10	0	2	1	N	Putative membrane/hyp/exported	At a rearranged genomic location (same as IchFam15)
IchFam20	11	6	0	1	0	0	1	Y	Putative membrane	
IchFam21	24	10	3	6	3	0	1	Y	Putative membrane	Interrupted by IchFam1
IchFam22	47	25	1	199	94	14	1	N	Coiled coil/hypothetical	
IchFam23	116	51	5	0			5	Y	Putative membrane/hypothetical	May share ancestor with IchFam25
IchFam24	0			65	44	0	1	Y	Putative membrane/hypothetical	
IchFam25	0			30	12	1	2	N	Putative membrane/hypothetical	May share ancestor with IchFam23

**Fig. 5.—:**
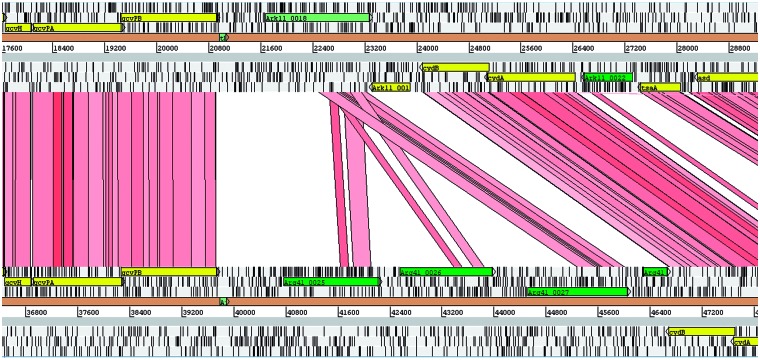
IchFam16 in the genome of *Ca*. I. hellenicum 2013Ark11B compared with *Ca*. I. sparus 2013Arg41. A single copy gene within this family is found in the genome of 2013Ark11B (shown above; Ark11_0018), expanded into three copies at the same location in the genome of 2013Arg41 (shown below; Arg41_0025-0027). The genomes were compared using TBLASTX and visualized using ACT (Carver et al. 2005). The six frames of translation are shown, with stop codons marked as black lines. Red bars between the genomes indicate amino acid identity (30–60%), which get paler as identity falls.

The vast majority of these families occur in tandem arrays (figs. 2 and supplementary S6, Supplementary Material online), with up to 47 adjacent members present on a single scaffold (IchFam22 in 2013Ark11B). The genome scaffolds have been rearranged to reflect this. In some cases, the families appear to occur in multiple arrays, which may reflect genome rearrangement or incorrect scaffold placement. An example of a family expansion from a single copy in the genome of 2013Ark11B to three copies in the genome of 2013Arg41 is shown in fig. 5. Tandem gene duplication and subsequent diversification appear to play a major role in this genus. Occasionally there are strand switches within a gene family array, and occasionally single family members are found separate from the rest of the array, indicating that genome rearrangement can result in family member displacement. Several families (IchFam7, 15, and 19) are found around a genome rearrangement which has occurred between the genomes of 2013Arg41 and 2013Ark11B which may have a mechanistic implication. RecA-dependent and RecA-independent processes such as slippage during DNA replication or repair have been suggested as mechanisms in the amplification of genes in eubacteria, as well as rolling circle amplification (Andersson and Hughes 2009). These *Ca*. Ichthyocystis genomes possess intact copies of *recA*. As mentioned above, the genome drafts contain a CDS encoding XerC family Phage integrase (*Ark11_1163/Arg41_1271*), as well as CDSs encoding both subunits of the phage-related integration host factor complex, IhfA (*Ark11_1167/Arg41_1264*) and IhfB (*Ark11_1186/Arg41_1372*), which are located in core parts of the genome, but at a distance from each other. Whether these are involved in the generation of the gene families will have to await further work.

**Fig. 6.—:**
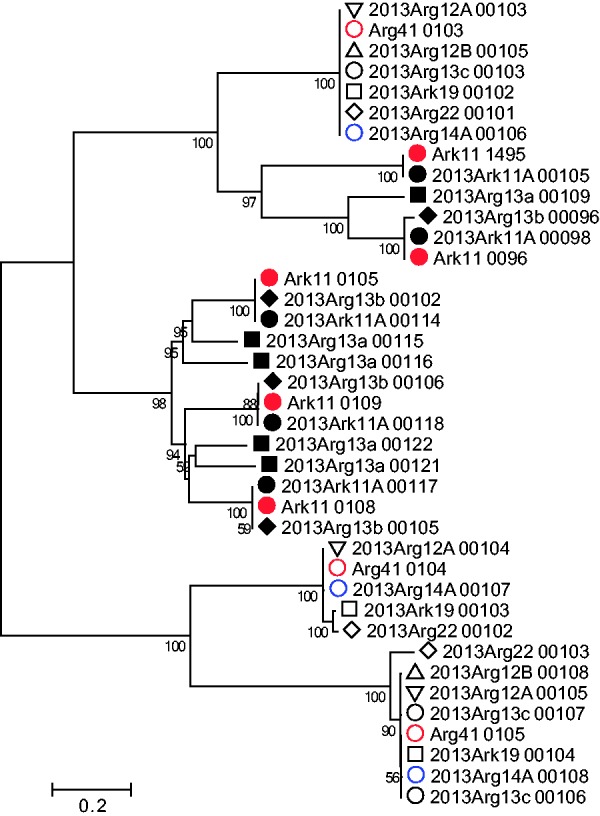
Unrooted maximum-likelihood tree of nucleotide sequences of members of gene family IchFam18. Labeling indicates the locus tag of the relevant CDS for the each draft genome, based on the ordered scaffolds, with filled symbols showing *Ca*. I. hellenium, open symbols showing *Ca*. I. sparus, and red used to color the two manually curated reference genome drafts. It is clear that duplication and diversification occurred prespeciation and is ongoing. Scale bar indicates number of substitutions per site and the bootstraps (100) shown as a percentage.

The phylogenetic trees of these families show that more closely related genomes share more closely related family members (fig. 6), and the duplication-diversification process appears to be ongoing (supplementary fig. S7, Supplementary Material online). In many cases, the genome drafts belonging to each species share the same family members, indicating that little diversification has occurred since these strains diverged (fig. 6); however, diversity can be seen in some cases indicating that the process is ongoing even over these timescales. In some cases, it can be seen that adjacent genes within one genome are the most similar, again emphasizing the tandem nature of the duplications (supplementary fig. S8, Supplementary Material online). In cases where there are family members in both species, the phylogenies can help to show the number of copies in the MRCA and how diversification has progressed since then (supplementary fig. S9, Supplementary Material online). It is difficult to speculate on the source of the families which occur only in one species.

This mechanism of duplication and diversification has been seen in other intracellular bacteria, including the ankyrin repeat domain T4SS effectors in *Wolbachia* (Siozios et al. 2013), and surface proteins in other species (Seubert et al. 2003; Toft and Andersson 2010). However, none of these studies show anything on the scale of what we see in *Ca*. Ichthyocystis, with almost a third of the genome, made up by these accessory features (table 3). *O. tsutsugamushi*, the obligate intracellular bacterium causing scrub typhus disease in animals and humans, has a huge proportion of repetitive DNA in the genome (46.5%), but all related to horizontally transferred DNA rather than CDSs with putative virulence functions (Nakayama et al. 2008).

### Functions of Gene Families

Manual annotation and further exhaustive analysis give very few clues to the putative functions of most of the gene families. The vast majority of gene family proteins are annotated as hypothetical, putative membrane proteins, or coiled-coil proteins (table 4), the latter being involved in structure or DNA binding. IchFam1 is the only family with identifiable functional domains conserved throughout the family, with many members containing ShET2 enterotoxin N-terminal domains (Pfam PF07906) linked to an extended region of 6–20 ankyrin repeats (ANK; Pfam PF00023) (supplementary fig. S10, Supplementary Material online). ANK repeat containing proteins are found throughout nature, from humans to bacteria (Jernigan and Bordenstein 2014). They are especially enriched in intracellular bacteria, with more ANK repeat containing proteins than free-living bacteria, and more ANK repeats per protein (Jernigan and Bordenstein 2014). The repeats encode a well conserved 33 amino acid domain making up two antiparallel alpha-helices linked by a loop, with a further loop linking one domain to the next. Human ankyrin contains 24 repeats, which build an elongated solenoid structure with an inner grove generating multiple protein-ligand binding sites (Wang et al. 2014), with the minimal site comprising 3–5 repeats. This would indicate that the members of IchFam1 would each be able to bind at least one ligand, presumably targeting the associated toxin domain to specific structures in the host cell. The presence of this extensive family in *Ca.* I. sparus, while being completely absent from *Ca.* I. hellenicum, is intriguing and one of the many fascinating questions to be answered through further studies. IchFam11 members also have repetitive motifs with some similarity to proteins involved in modulating host cell signaling (Lin et al. 2006) or adhesion (Varney et al. 2002), and several other families contain members with apparent repetitive motifs (IchFam2, 22, 23, 25) with unknown functions.

Effective was used to predict the number of putative T3SS effectors among the gene family members. Over half of the members of most gene families were designated as containing predicted T3SS secretion signals, with T4SS effectors also predicted (table 4; note that many of the family members on smaller scaffolds cannot be scanned in this way as they lack assembled N and C termini), indicating that these have key virulence functions. In contrast to most obligate intracellular bacteria, gene families have been described within *Neochlamydia* and related bacteria, members of which are also putative T3SS effectors (Domman et al. 2014).

While in many cases the duplicated genes are of similar sizes, in other cases more fragmented versions can be seen, implying that this duplicated version did not diversify to encode a functionally useful protein, and has thus degraded to form a pseudogene. These have not been annotated as pseudogenes, as lack of function cannot currently be confirmed. A curious but notable example of this effect is the duplication and degradation of an enolase gene in the genome of 2013Arg41, which appears to have been duplicated with members of IchFam24 and can be seen in several pseudogenized forms through the genome. These make up the vast majority of pseudogenes in this genome and show how drift relentlessly removes unnecessary genes. This gives strong evidence that these gene families are indeed functionally important for the lifestyle of these bacteria.

It would be fascinating to attempt to correlate the presence of alternative gene families in the two *Ca*. Ichthyocystis species with the phenotypic differences observed microscopically (Seth-Smith, et al. 2016), but with so little functional information this is currently impossible.

## Conclusion

We present a thorough genomic study on some fascinating novel pathogenic bacteria. Eleven genome drafts have been constructed from preserved material, in the absence of cultured strains, with optimized assembly, manual annotation, and detailed analysis. The genomes of the novel bacteria within genus *Ca*. Ichthyocystis illustrate novel evolutionary mechanisms for obligate intracellular pathogens. The metabolic capacity of these bacteria shows that they cannot grow independently, as they are unable to synthesize any amino acids. This has implications for how they transmit, which is unknown, and provides information which could also aid attempts at *in vitro* culture (Omsland et al. 2008). The evolution of the massively expanded gene families implies that the members of these have critical functions and are probably selected for as virulence factors. Several other factors implicated in virulence are present, including T2SS, T3SS, T4SS, and Tfp, and the cell wall structure of these bacteria is unusual as it seems to lack LPS. In order to gain further insights, in the absence of the ability to culture these organisms, we will attempt to look at the transcriptome and use further imaging or model organisms to investigate the host–pathogen relationship.

## Supplementary Material

Supplementary tables S1–S7 and figs. S1–S10 are available at *Genome Biology and Evolution* online (http://www.gbe.oxfordjournals.org/).

Supplementary Data
