# Supplementary material for: Host-Associated Genomic Features of the Novel Uncultured Intracellular Pathogen Ca. Ichthyocystis Revealed by Direct Sequencing of Epitheliocysts
Source: Genome Biol Evol. 2016 May 10;8(6):1672–89. doi: 10.1093/gbe/evw111 (PMC4943182; doi:10.1093/gbe/evw111)
Supplement: Supplementary Data [file supp_8_6_1672__index.html]

Host-Associated Genomic Features of the Novel Uncultured Intracellular Pathogen Ca. Ichthyocystis Revealed by Direct Sequencing of Epitheliocysts — Supplementary Data 

# Host-Associated Genomic Features of the Novel Uncultured Intracellular Pathogen *Ca.* Ichthyocystis Revealed by Direct Sequencing of Epitheliocysts

## Supplementary Data

files

- Supplementary Data - docx file
- Supplementary Data - xlsx file
