# Supplementary material for: Host-Associated Genomic Features of the Novel Uncultured Intracellular Pathogen Ca. Ichthyocystis Revealed by Direct Sequencing of Epitheliocysts
Source: Genome Biol Evol. 2016 May 10;8(6):1672–89. doi: 10.1093/gbe/evw111 (PMC4943182; doi:10.1093/gbe/evw111)
Supplement: Supplementary Data [file supp_evw111_Suppl_figures_070416b.docx]

| 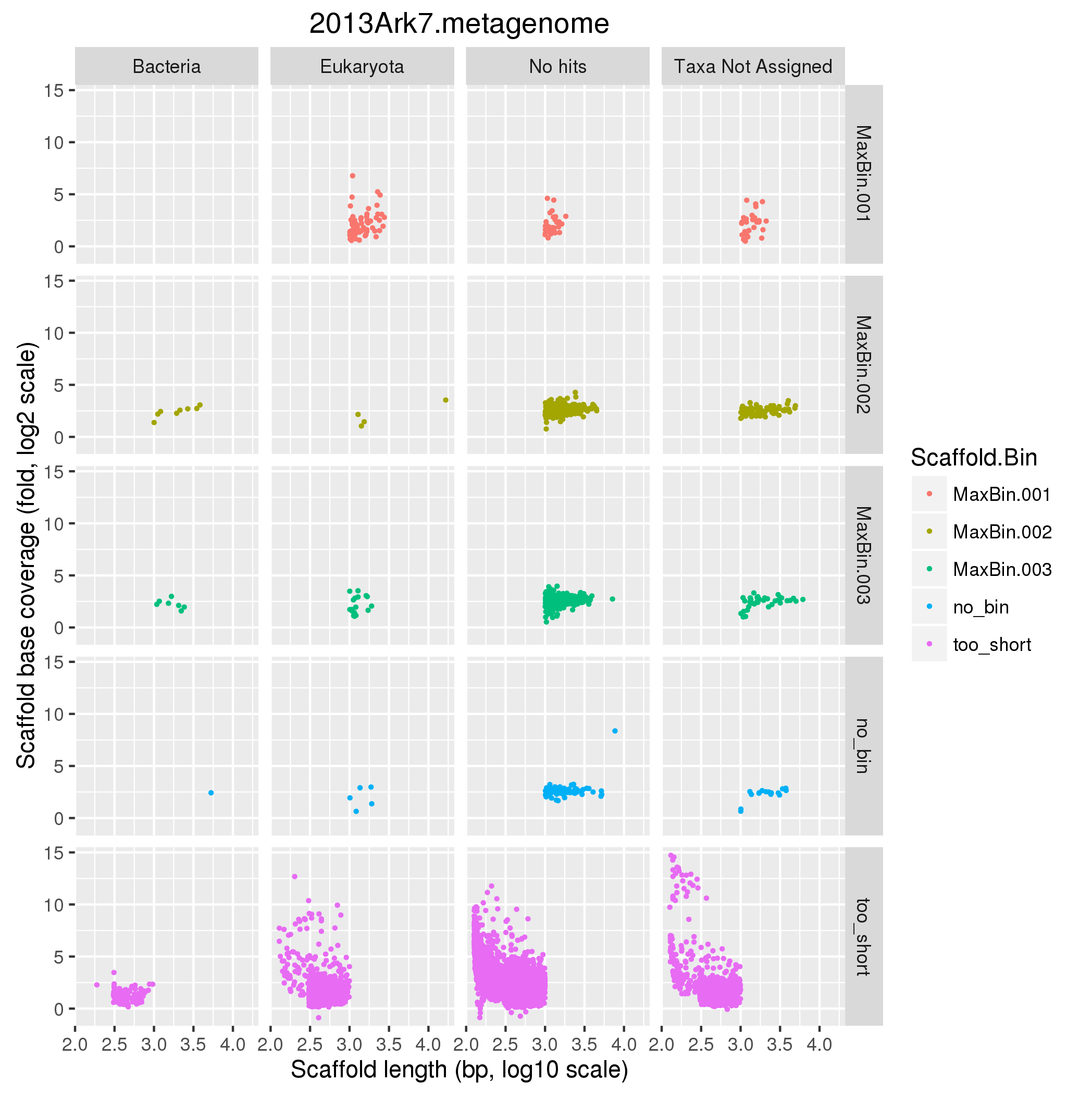 | 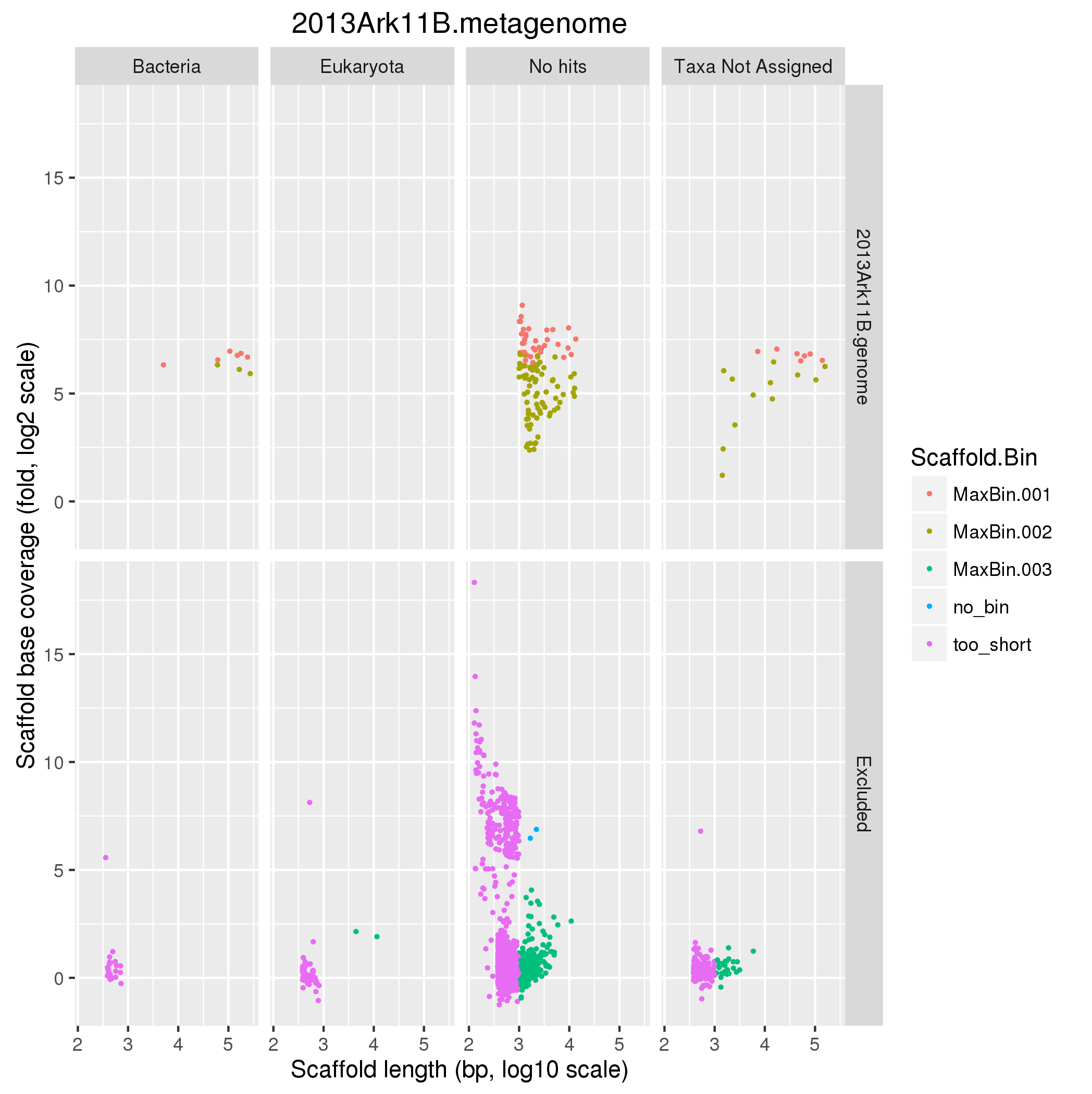 |
| --- | --- |

| 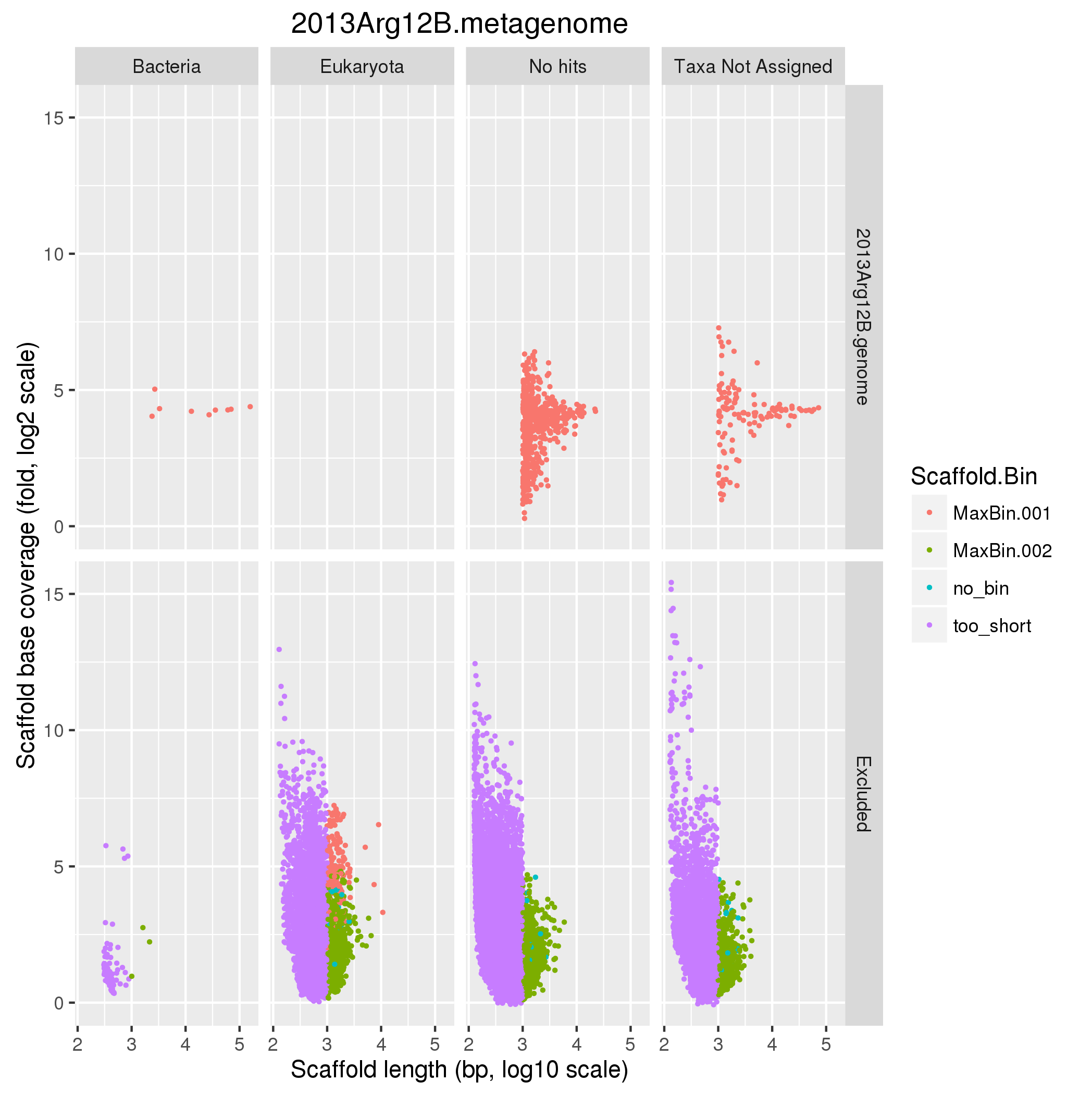 | 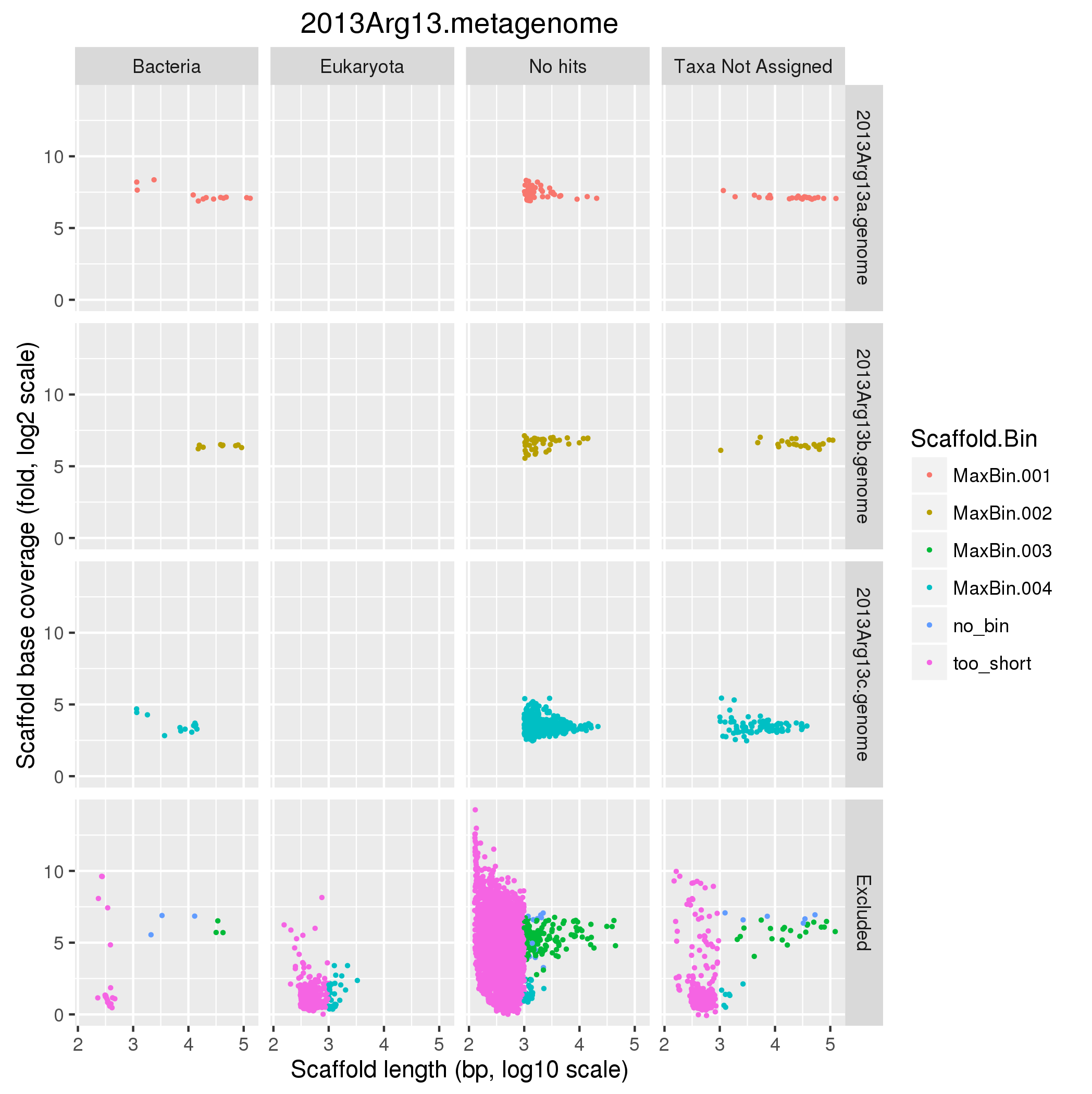 |
| --- | --- |

**Figure S1. Constructing genome drafts from cyst mini-metagenomes with estimated bacterial genome diversity of 2 and above.** Each mini-metagenome is shown in one multi-panel plot, where the rows are recovered genome drafts or scaffold bins and columns are taxonomic origins. Within each panel, the x axis represents scaffold length in log10 scale, the y axis represents scaffold coverage in log2 scale. Each dot represents one scaffold, coloured by scaffold bins as defined by MaxBin (Wu, et al. 2014), where “no_bin” means scaffolds not assigned to any bins by the software and “too_short” means scaffolds shorter than 1 Kb thus not subjected to binning by MaxBin. Scaffolds were assigned to taxonomic groups using MEGAN (Huson, et al. 2011) based on BLAST (Altschul, et al. 1990) comparisons against the NCBI non-redundant nucleotide database (see Materials and Methods for details). “No hits” means that BLAST comparisons did not find any hits in the database (e value cutoff= 10), while “Taxa Not Assigned” means no taxonomic group was assigned by MEGAN, mainly due to BLAST hits with bit scores lower than the cutoff value applied (200). The genome drafts of 2013Arg13a,b,c and 2013Arg12B were each recovered from single scaffold bins in which the set of bacterial marker genes was rather complete. The genome draft of 2013Ark11B was reconstructed from two scaffold bins to maximize the completeness and uniqueness of bacterial marker genes. Scaffold bins in 2013Ark7 harboured too few bacterial marker genes, and eukaryotic scaffolds were scattered in all bins, thus no pathogen genome was recovered (Table S2).

| **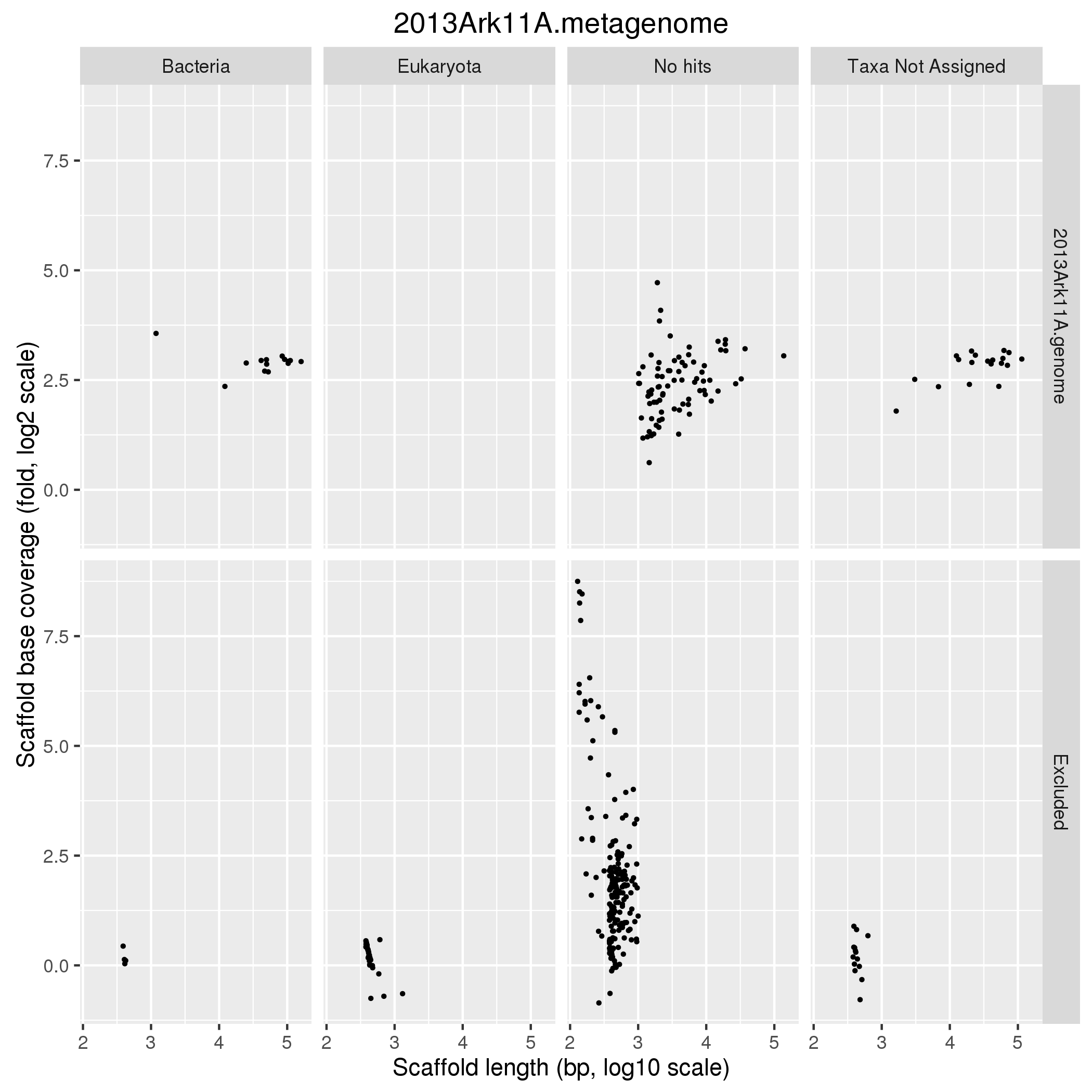** | **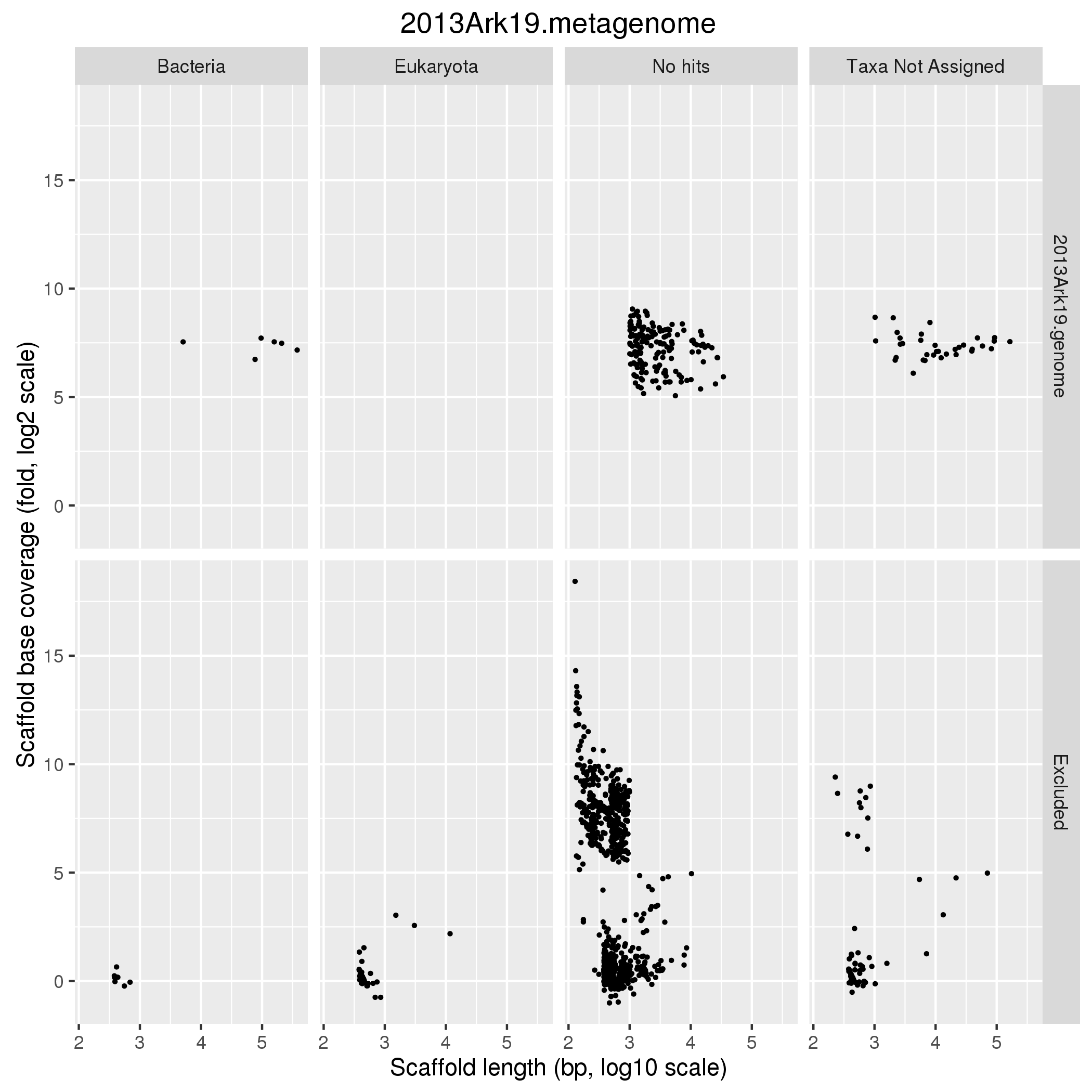** |
| --- | --- |
| **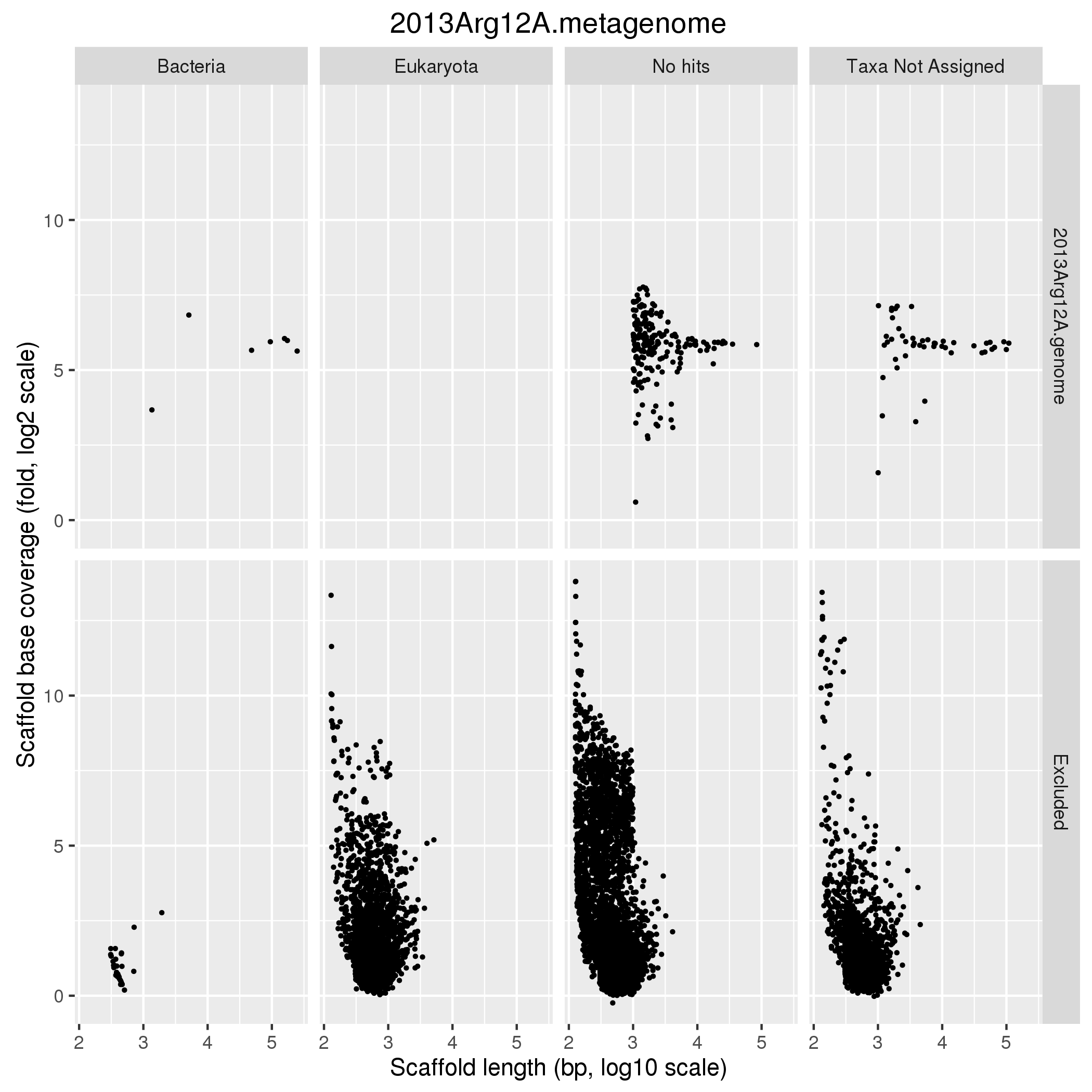** | **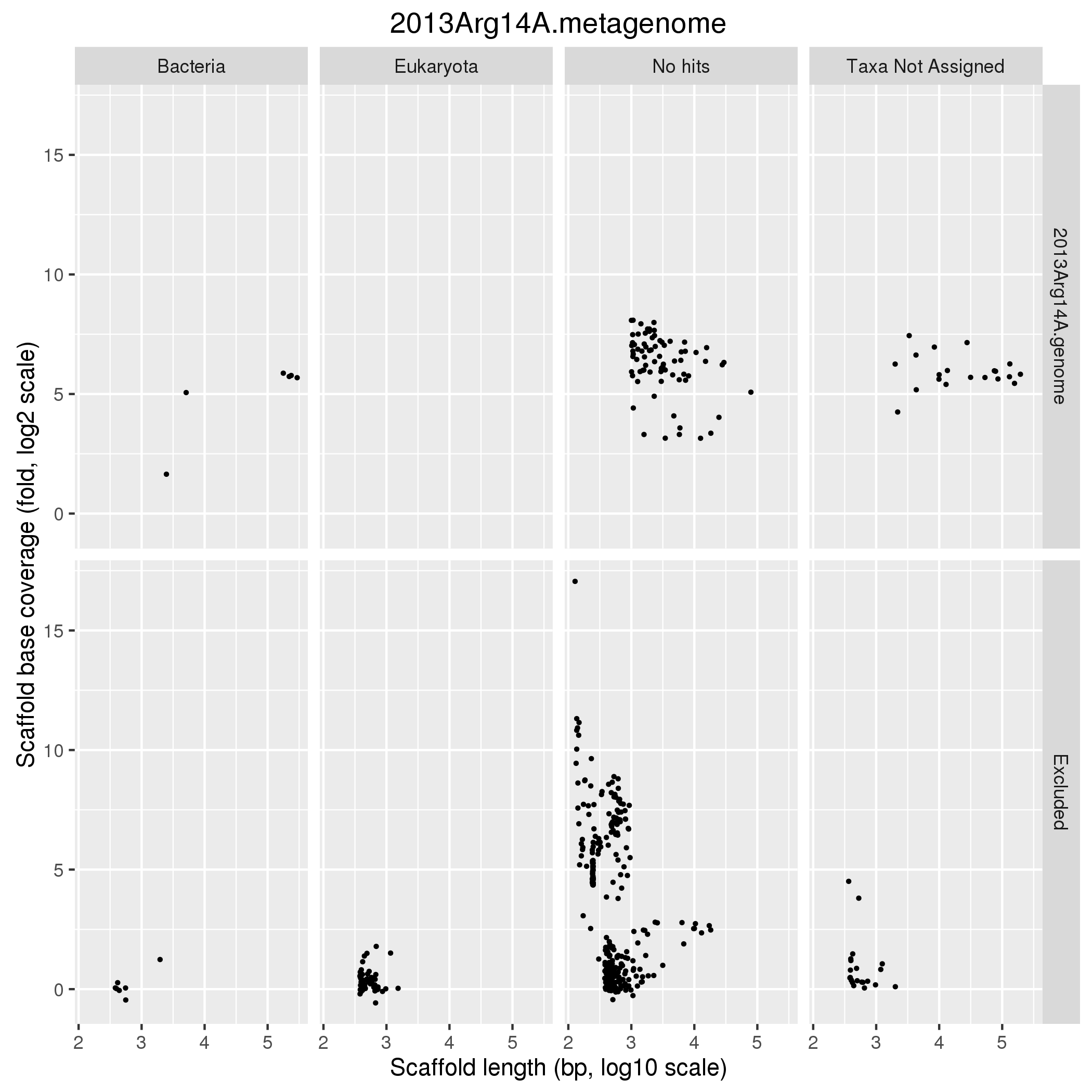** |
| **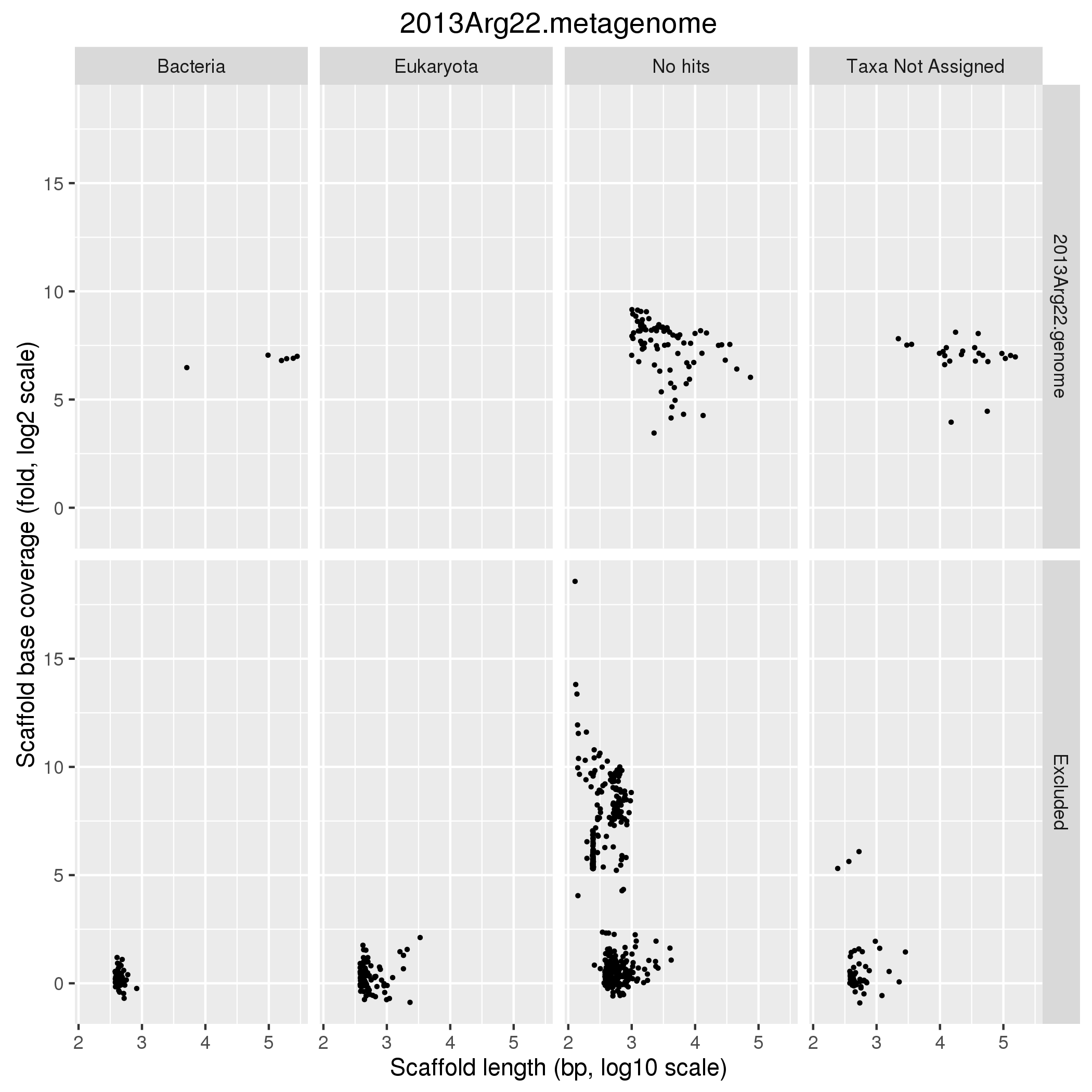** | **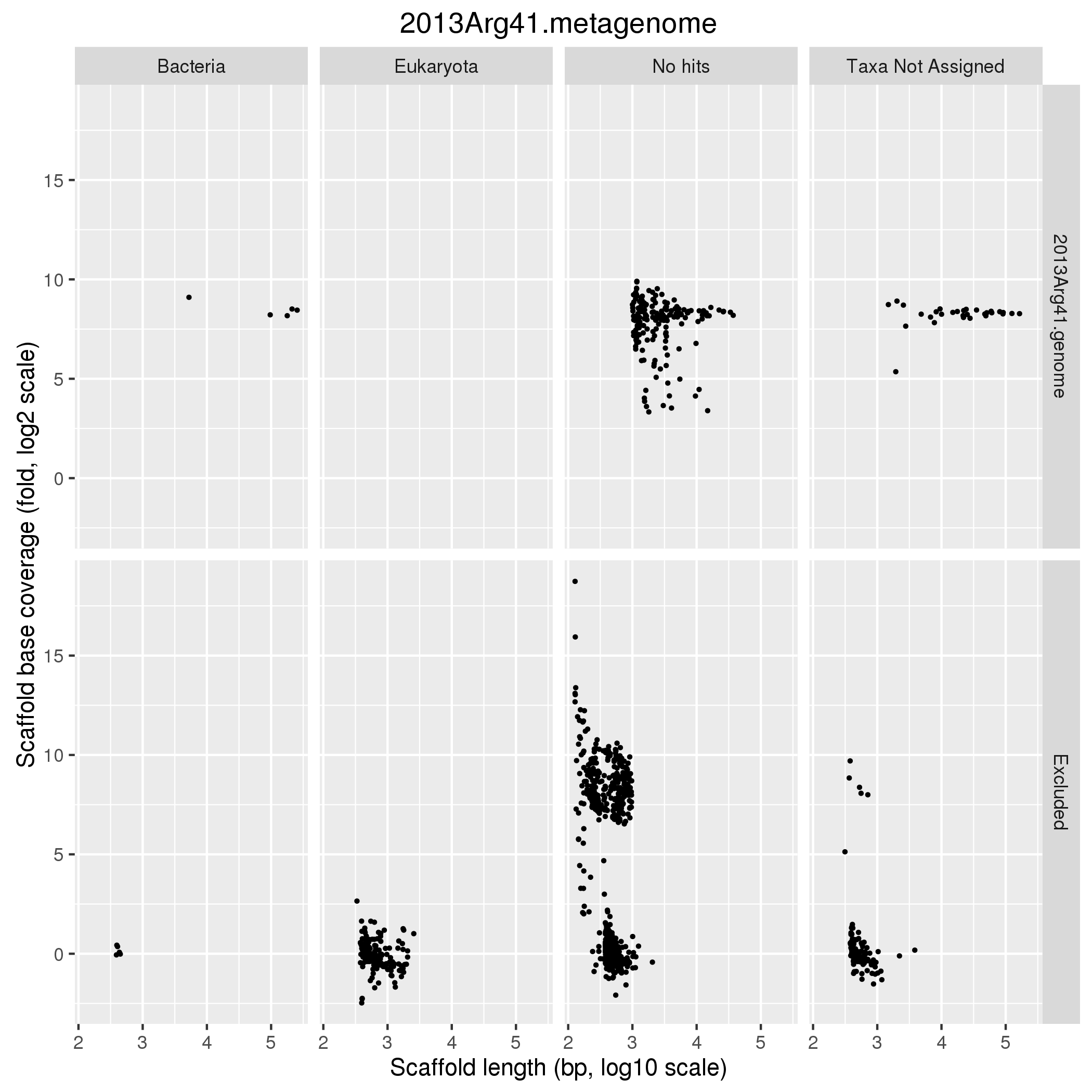** |

**Figure S2. Constructing genome drafts from cyst mini-metagenomes with estimated bacterial genome diversity of 1.** Each mini-metagenome is shown using one multi-panel plot, where the rows are recovered genome drafts or scaffold bins and columns are taxonomic origins. Within each panel, the x axis represents scaffold length in log10 scale, the y axis represents scaffold coverage in log2 scale. Scaffolds were assigned to taxonomic groups as above, using MEGAN based on BLAST comparisons against the NCBI non-redundant nucleotide database. “No hits” means that BLAST comparisons did not find any hits in the database (e value cutoff = 10), while “Taxa Not Assigned” means no taxonomic group was assigned by MEGAN, mainly due to BLAST hits with bit scores lower than the cutoff value applied (200). The scaffold length cutoff applied was 1 Kb. The scaffold coverage thresholds applied were: 8x for 2013Arg14A and 2013Ark11A, 16x for 2013Arg22 and 2014Arg41, 32x for 2013Ark19. For 2013Arg12A, eukaryotic sequences were removed by sequence similarity to known sequences because there was no good separation using a coverage threshold.


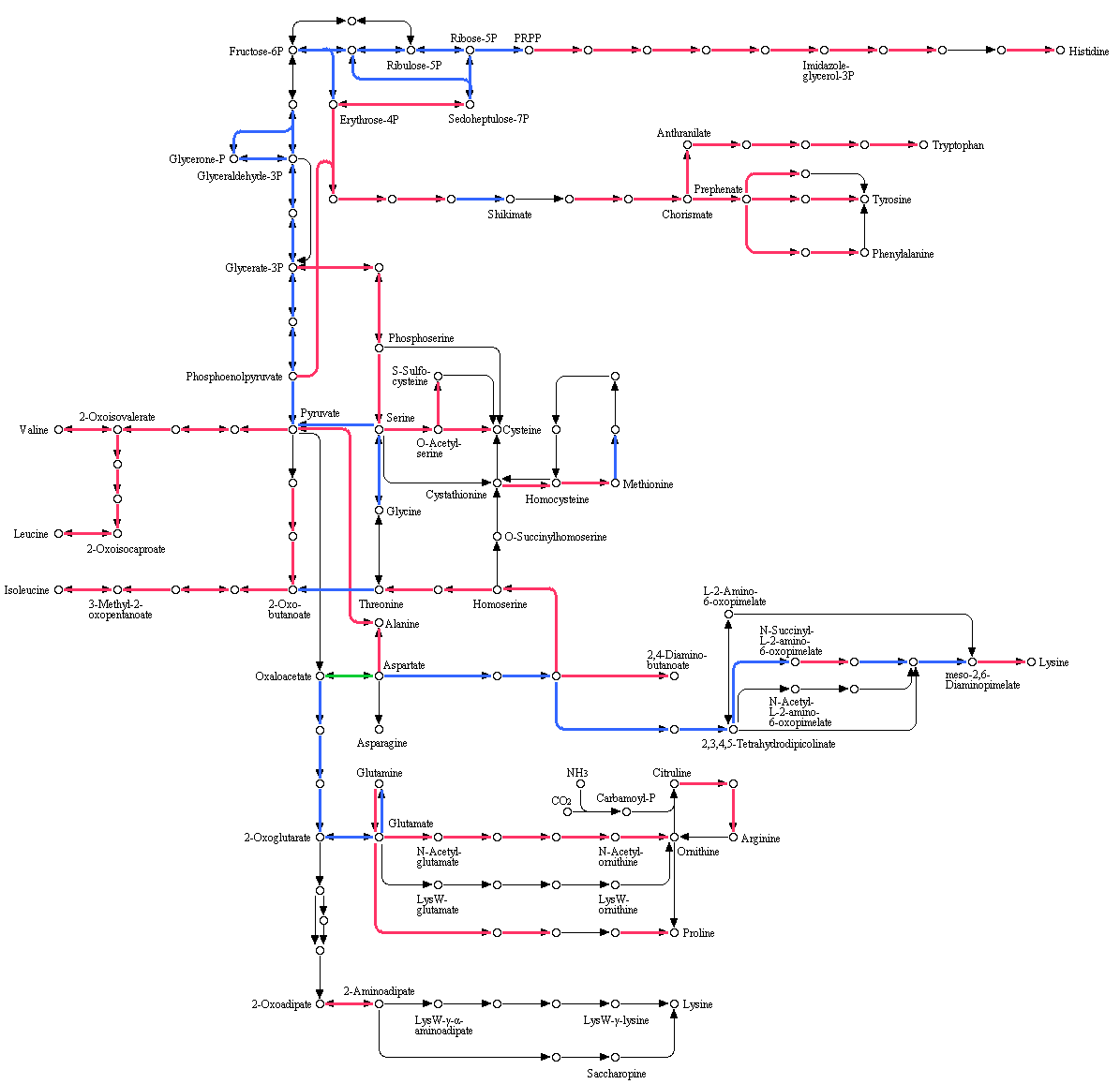


**Figure S3. Amino acid synthesis pathways in *Ca*. Ichthyocystis genome drafts in comparison to those in the genome of free-living beta-proteobacteria *Janthinobacterium* sp. Marseille.** Pathway components present only in. *J.* sp. Marseille were coloured in red, those found only in *Ca*. Ichthyocystis in green, in both *Ca*. Ichthyocystis and *J.* sp. Marseille were coloured in blue. While *J.* sp. Marseille is capable of synthesizing different amino acids, none of the amino acid synthesis pathways are complete in any of the *Ca*. Ichthyocystis genomes.


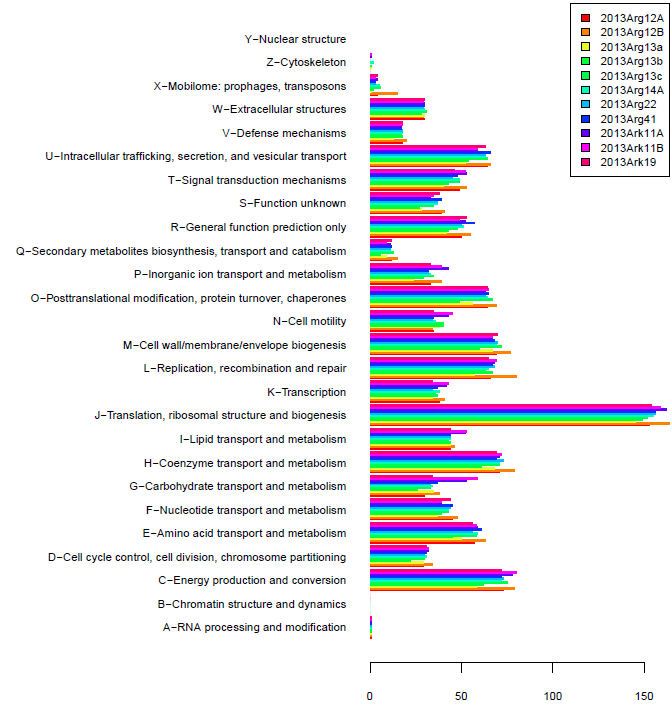


**Figure S4. COG analysis of *Ca*. Ichthyocystis genus.** Comparison of the numbers of COG families (x axis) predicted from the gene models extracted from the 11 *Ca*. Ichthyocystis genome drafts.


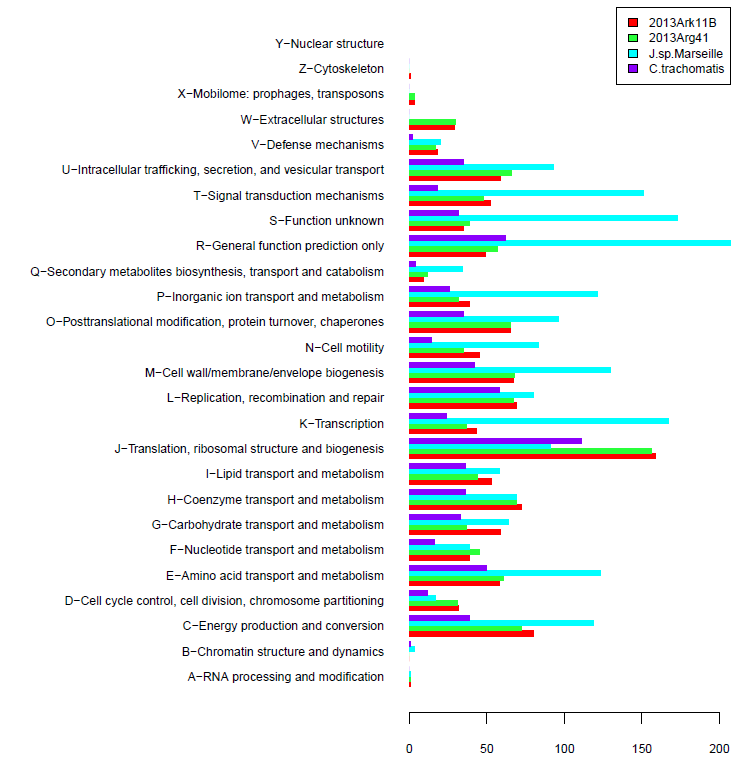


**Figure S5. Clusters of orthologous groups** (**COG) analysis of *Ca*. Ichthyocystis compared against other genomes.** Comparison of the numbers of COG families (x axis) predicted from the CDSs in the two reference genomes against those in the the free living beta-proteobacterium *Janthinobacterium* sp. Marseille and the intracellular pathogen *Chlamydia trachomatis*.

**
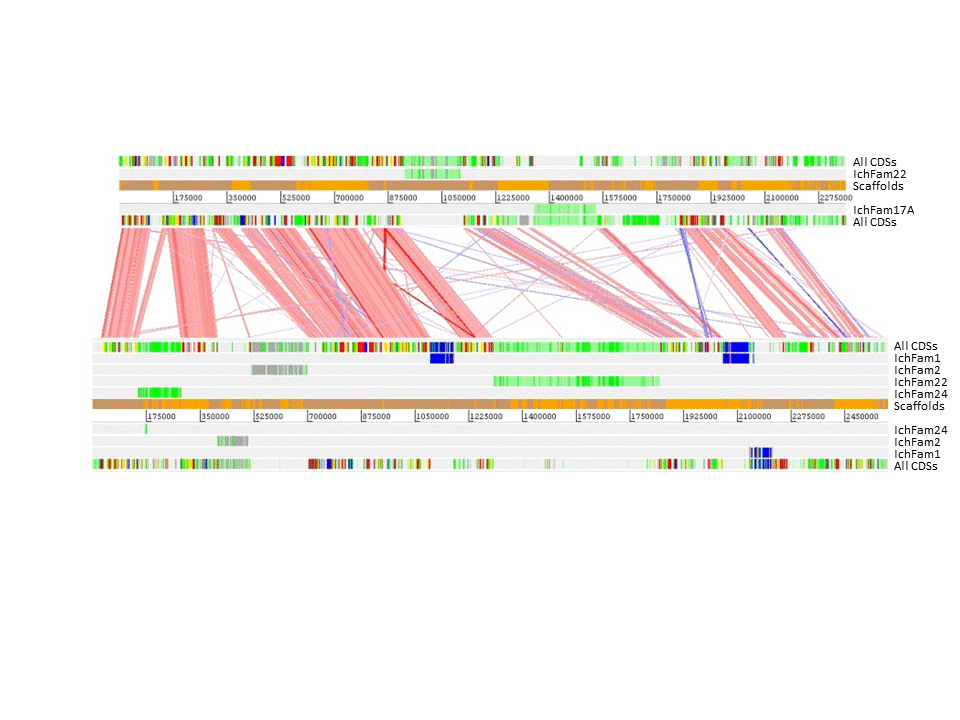
**

**Figure S6. Large gene families in *Ca*. I. hellenicum 2013Ark11 and *Ca*. I. sparus 2013Arg41.** Draft genomes of 2013Ark11 (top) and 2013Arg41 (bottom) were compared using tblastx and visualised using ACT. Red bars between the genomes indicate amino acid identity (30-60%), which get paler as identity falls. Blue bars indicate inversions. Sections with no matches indicate regions of difference between the genomes. Orange and brown stripes along the genome indicate the scaffolds, numbered in bp along the genome. Each grey line shows manually annotated CDSs in forward (above scaffolds) and reverse (below scaffolds) frames with colour coding for CDS functions: dark blue for pathogenicity/adaptation, black for energy metabolism, red for information transfer, dark green for surface associated, yellow for central/intermediary metabolism, pale green for hypothetical proteins, pale blue for regulators, orange for conserved hypothetical, brown for pseudogenes, pink for phage and IS elements, and gray for miscellaneous. The tracks presented include, as indicated: all annotated CDSs, the identified members of families IchFam17A and IchFam22 (2013Ark11) and IchFam1, IchFam2, IchFam22 and IchFam24 (2013Arg41), in the relevant frames. It is clear that these gene families occur in large arrays, two in the case of IchFam1, often only on one strand.

**
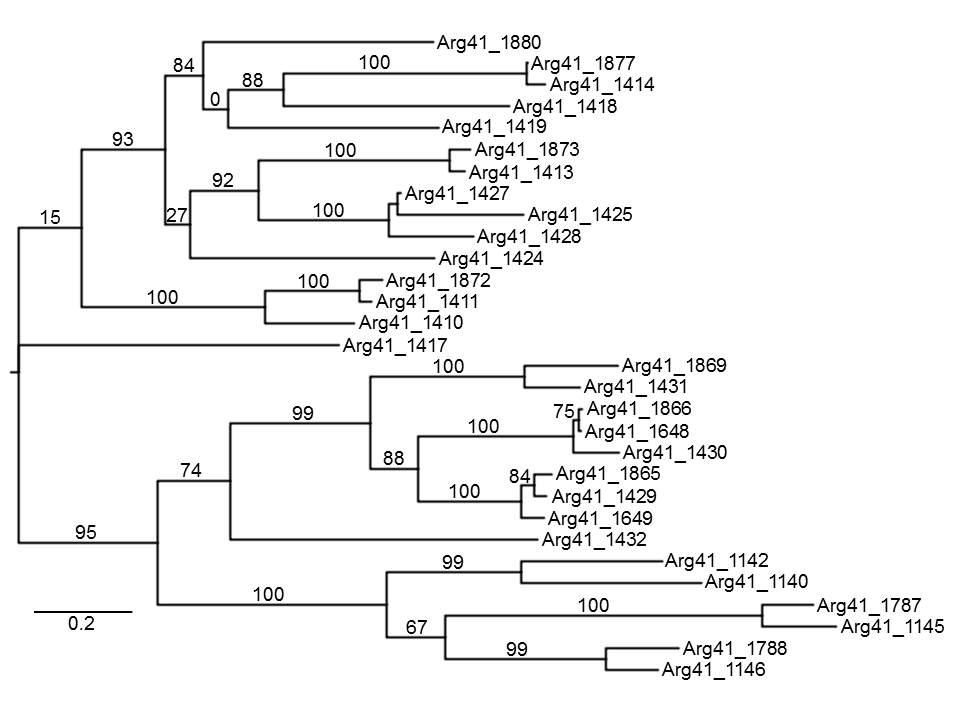
**

**Figure S7**. **Phylogenetic tree of IchFam25 belonging to 2013Arg41.** Phylogeny based on nucleotide sequences aligned with clustalo, with tree created in PhyML with 100 bootstraps. Ongoing duplication and diversification is indicated by the shape of the tree. Scale bar indicates number of substitutions per site.


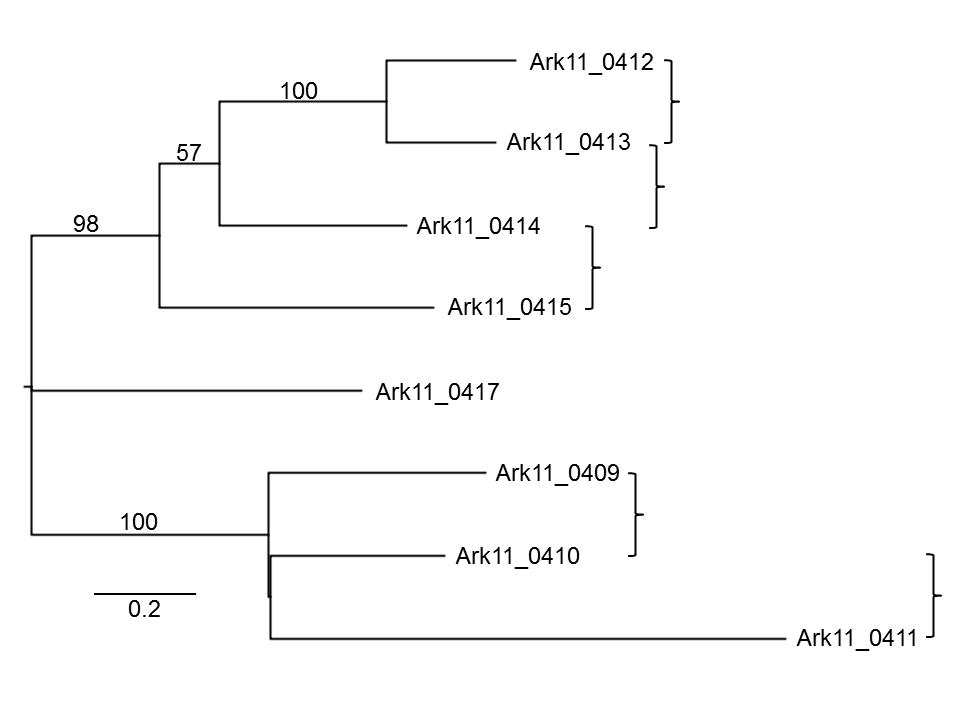


**Figure S8. Phylogenetic tree of IchFam9 belonging to 2013Ark11, which are all assembled on a single scaffold.** Phylogeny based on nucleotide sequences aligned with clustalo, with tree created in PhyML with 100 bootstraps. Clear incidences of recent duplication of adjacent genes are shown with brackets. Scale bar indicates number of substitutions per site.


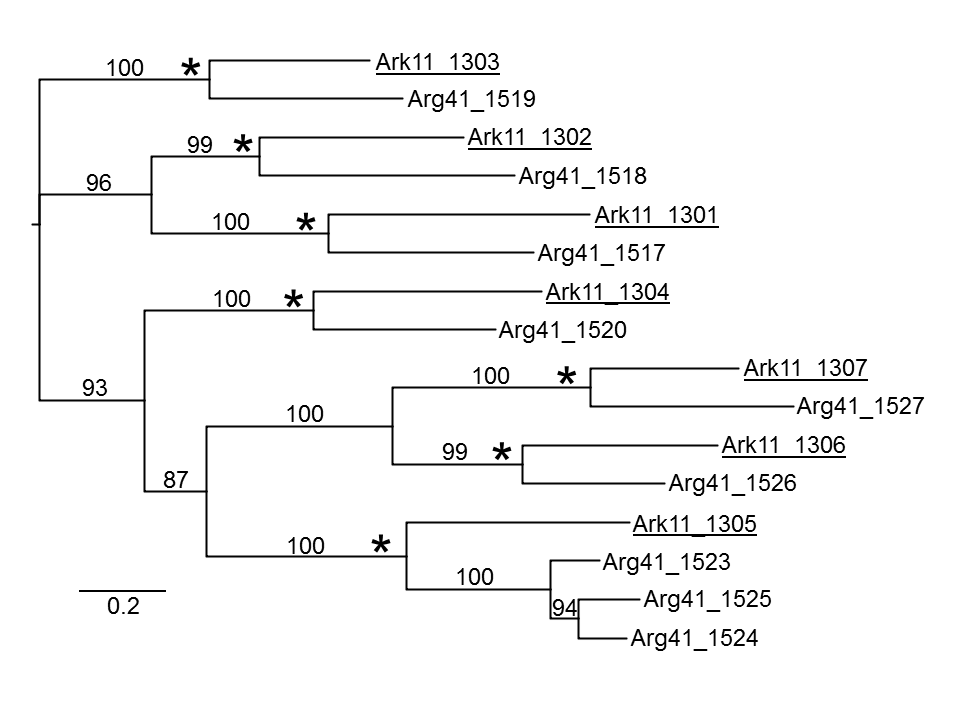


**Figure S9. Phylogenetic tree of IchFam19 within 2013Ark11 and 2013Arg41**. Phylogeny based on nucleotide sequences aligned with clustalo, with tree created in PhyML with 100 bootstraps. The most recent common ancestor (MRCA) of the two strains appears to have contained 7 members of this family (asterisks), which have diverged in the two strains, and one member has duplicated further within the genome of 2013Arg41 (bottommost clade). Gene family members belonging to the genome of 2013Ark11 are underlined, the others belong to the genome of 2013Arg41. Scale bar indicates number of substitutions per site.

**
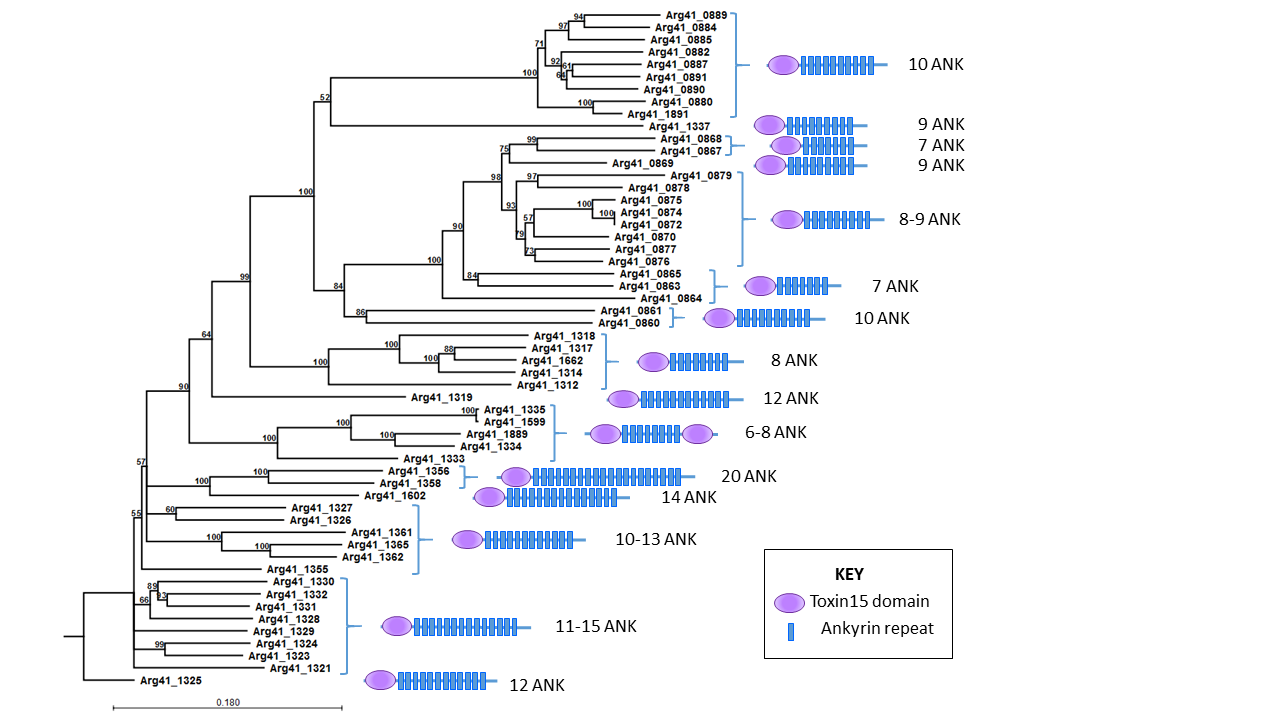
**

**Figure S10. Phylogeny and characteristics of IchFam1 from 2013Arg41**. Phylogeny based on amino acid sequences aligned with ClustalW, with unrooted neighbour joining tree created based on Maximum Likelihood using a Jukes-Cantor model and 1000 bootstraps. The minimum bootstrap setting was set to 50%. For this analysis, only full-length members of IchFam1 were selected, all of which contain an N-terminal toxin-domain. One clade is composed of five twin-toxin domain members. All other clades tend to group IchFam1 members into those with similar numbers of ANK repeats. Arg41_1356 and Arg41_1358 are the largest members, with an ANK-domain rivalling in size the 24 ANK-repeats of eukaryotic Ankyrin. The ANK-domain search was aided greatly by sequence comparison against a protein-structure database using Swissmodel (http://swissmodel.expasy.org). Scale bar indicates expected number of changes per amino acid.

**Supplementary References**

Altschul SF, Gish W, Miller W, Myers EW, Lipman DJ 1990. Basic local alignment search tool. Journal of Molecular Biology 215: 403-410.

Huson DH, Mitra S, Ruscheweyh H-J, Weber N, Schuster SC 2011. Integrative analysis of environmental sequences using MEGAN4. Genome Res 21: 1552-1560. doi: 10.1101/gr.120618.111

Wu Y-W, Tang Y-H, Tringe SG, Simmons BA, Singer SW 2014. MaxBin: an automated binning method to recover individual genomes from metagenomes using an expectation-maximization algorithm. Microbiome 2: 1-18. doi: 10.1186/2049-2618-2-26
